# Supplementary material for: BCL6 controls contact-dependent help delivery during follicular T-B cell interactions
Source: Immunity. 2021 Oct 12;54(10):2245–2255.e4. doi: 10.1016/j.immuni.2021.08.003 (PMC8528402; doi:10.1016/j.immuni.2021.08.003)
Supplement: Document S1. Figures S1–S7 [file mmc1.pdf]

Immunity, Volume *54*

Supplemental information

BCL6 controls contact-dependent help delivery during follicular T-B cell interactions

Dan Liu, Jiacong Yan, Jiahui Sun, Bo Liu, Weiwei Ma, Ye Li, Xingxing Shao, and Hai Qi

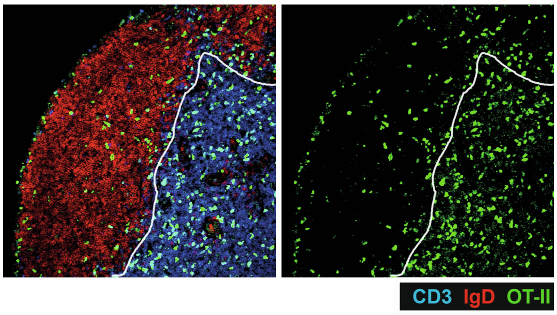

$$\text{Follicle Recruitment Index (FRI)} = \frac{\text{T-cell density in the follicle}}{\text{T-cell density in the T-zone}}$$

Figure S1. Definition of follicle recruitment index (FRI), related to Figure 1.

The follicle recruitment index (FRI) to quantify the efficiency of OT-II follicular localization. The experimental system and color code as in Figure 1B.

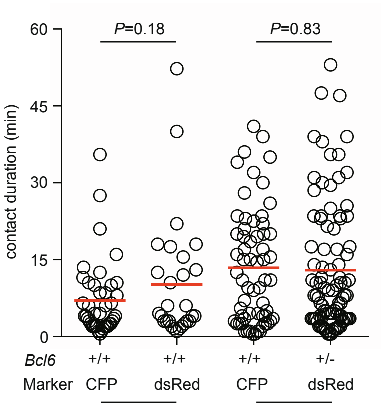

Figure S2. Comparable duration of contacts between MD4 B cells and *Bcl6*$^{+/+}$ or *Bcl6*$^{+/-}$ OT-II T cells at the T-B border, related to Figure 2.

B6 mice that received transfer of GFP-expressing MD4 B cells, control CFP-expressing *Bcl6*$^{+/+}$ and dsRed-expressing test *Bcl6*$^{+/-}$ or test *Bcl6*$^{+/+}$ OT-II T cells were immunized with HEL-OVA. Draining lymph nodes were intravitally imaged 36-48 hours later, a time point at which T-B contacts were mainly seen at the T-B border. Contact durations were quantitated (see corresponding Movie S1 and S2). Each dot represents one contact. Data pooled from two independent imaging experiments, each of which involved at least 2 recipient mice.

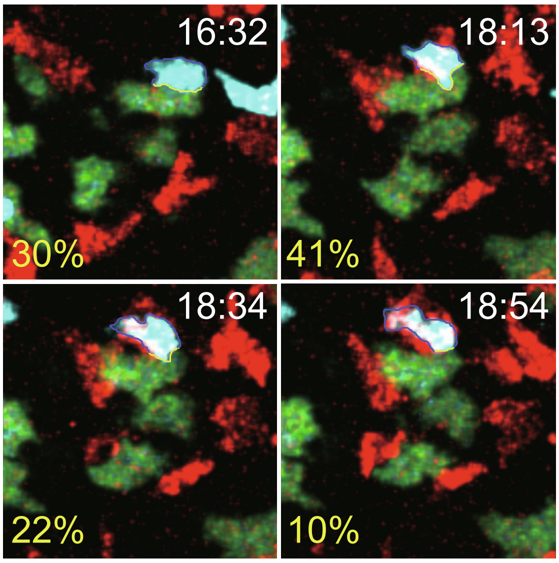

$$\mathrm{SEI} = \max\left(\frac{\text{Length of contact}}{\text{T-cell perimeter}}\right)$$

Figure S3. Definition of surface engagement index (SEI), related to Figure 2.

In maximum-intensity projection, the proportion of T-cell perimeter (blue) that is engaged by an interacting B cell (line fragment in yellow) is traced overtime and the maximum proportion reached during a continuous contact incidence is defined as the surface engagement index.

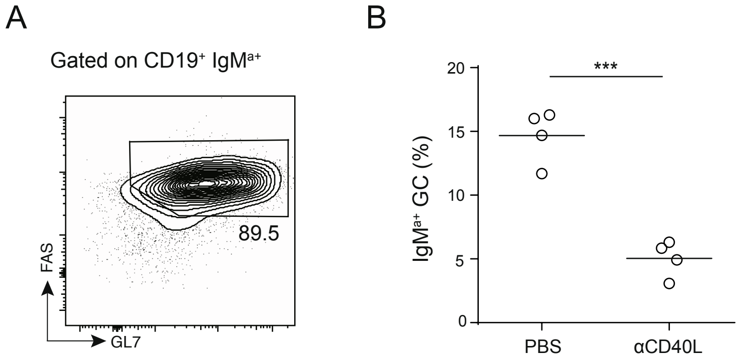

Figure S4. Follicular phase of CD40L delivery is required for GC formation, related to Figure 4.

B6 mice that received transfer of MD4 B cells and OT-II T cells were immunized with HEL-OVA and treated with αCD40L antibody (200 µg) or PBS 50-60 hours later. **A**, Representative FACS profile and frequency of FAS^{hi} $GL7^{hi}$ GC B cells in IgM^{a+} $CD19^{+}$ B cells at 5 days post immunization. **B**, Frequencies of IgM^{a+} MD4 GC cells in total $CD19^{+}$ B cells. Each symbol represents one mouse, and lines denote means. One of three experiments with similar results is shown. *** $P<0.001$.

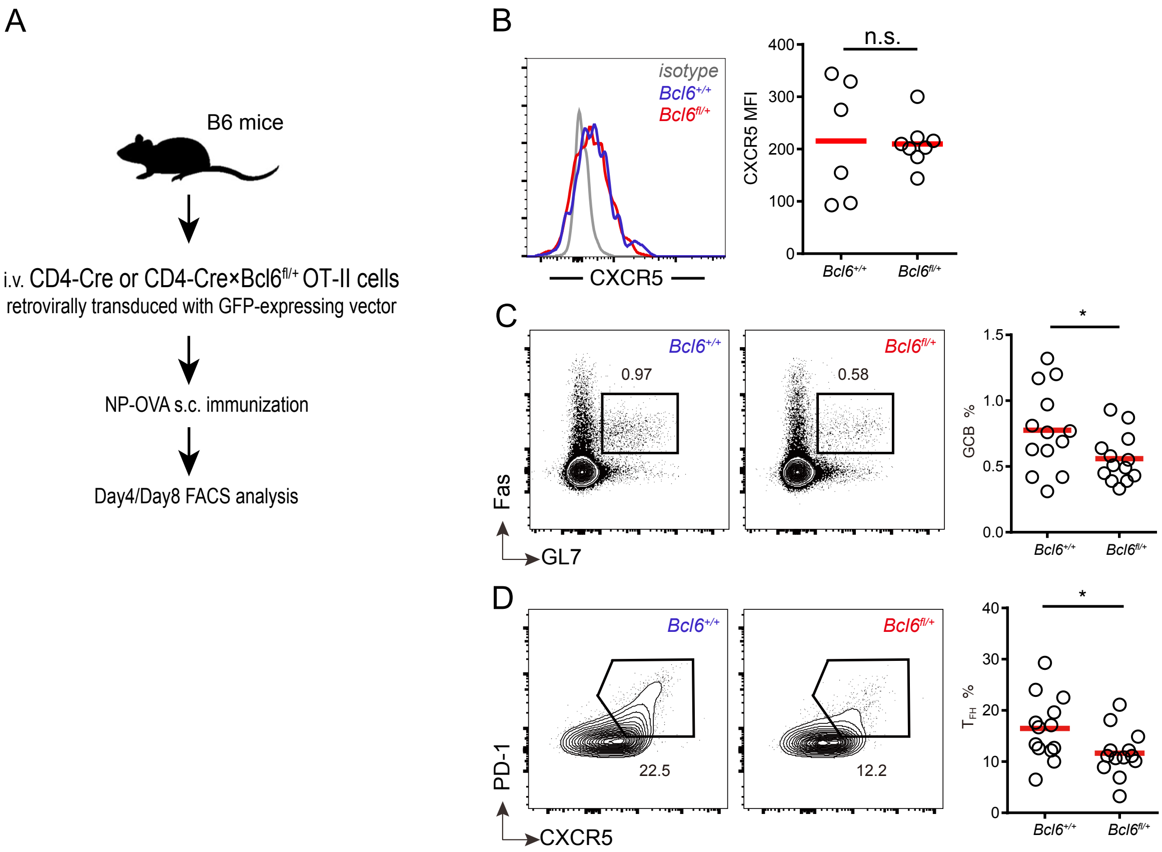

Figure S5. Defective helper functions due to BCL6 haploinsufficiency as demonstrated with T-cell-specific conditional BCL6 deficiency, related to Figure 5.

A, The experimental scheme. **B,** Representative histograms and MFIs of surface CXCR5 expression on OT-II T cells of indicated *Bcl6* genotypes in draining lymph nodes 4 days after NP-OVA immunization. **C,D,** Representative flow-cytometry profiles and frequencies of $FAS^{hi}GL7^{hi}$ GC B cells in total $B220^{+}$ B cells (**C**) or $CXCR5^{hi}PD\text{-}1^{hi}$ Tfh cells in OT-II T cells (**D**) 8 days after NP-OVA immunization in CD45.1 $Sap^{-/-}$ recipients. Each symbol represents one mouse, and lines denote the means. Data are pooled from 2 independent experiments. n.s., not significant. * $P<0.05$.

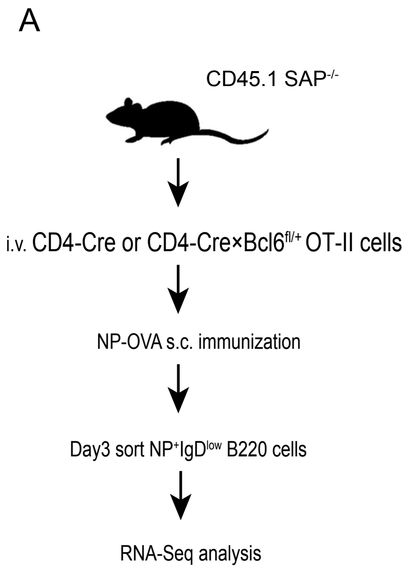

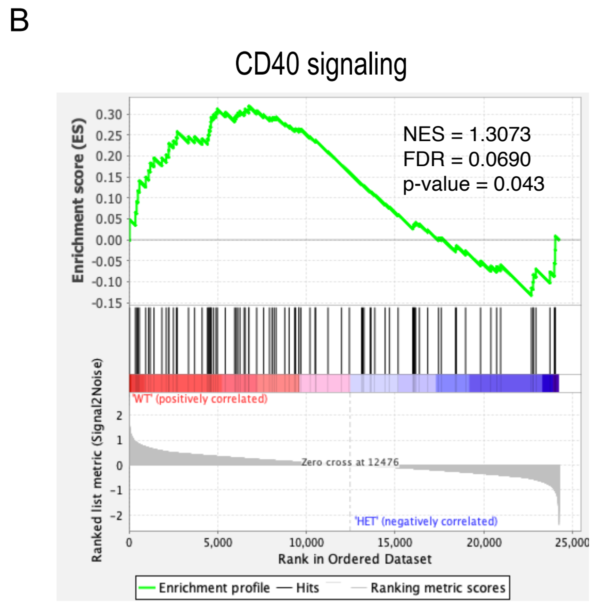

Figure S6. Reduced CD40 signaling signature in antigen-activated B cells helped by BCL6-insufficient T cells, related to Figure 5.

mRNA sequencing of pre-GC B cells helped by CD4-Crex*Bcl6*$^{fl/+}$ or CD4-Cre OT-II T cells. **A,** The experimental scheme. **B,** Enrichment analysis comparing pre-GC antigen-specific B cells helped by CD4-Crex*Bcl6*$^{fl/+}$ ("HET") or by CD4-Cre ("WT") OT-II T cells for a gene set upregulated by CD40L stimulation of Ramos cells previously reported (Basso et al., 2004). NES, normalized enrichment score; FDR, false discovery rate.

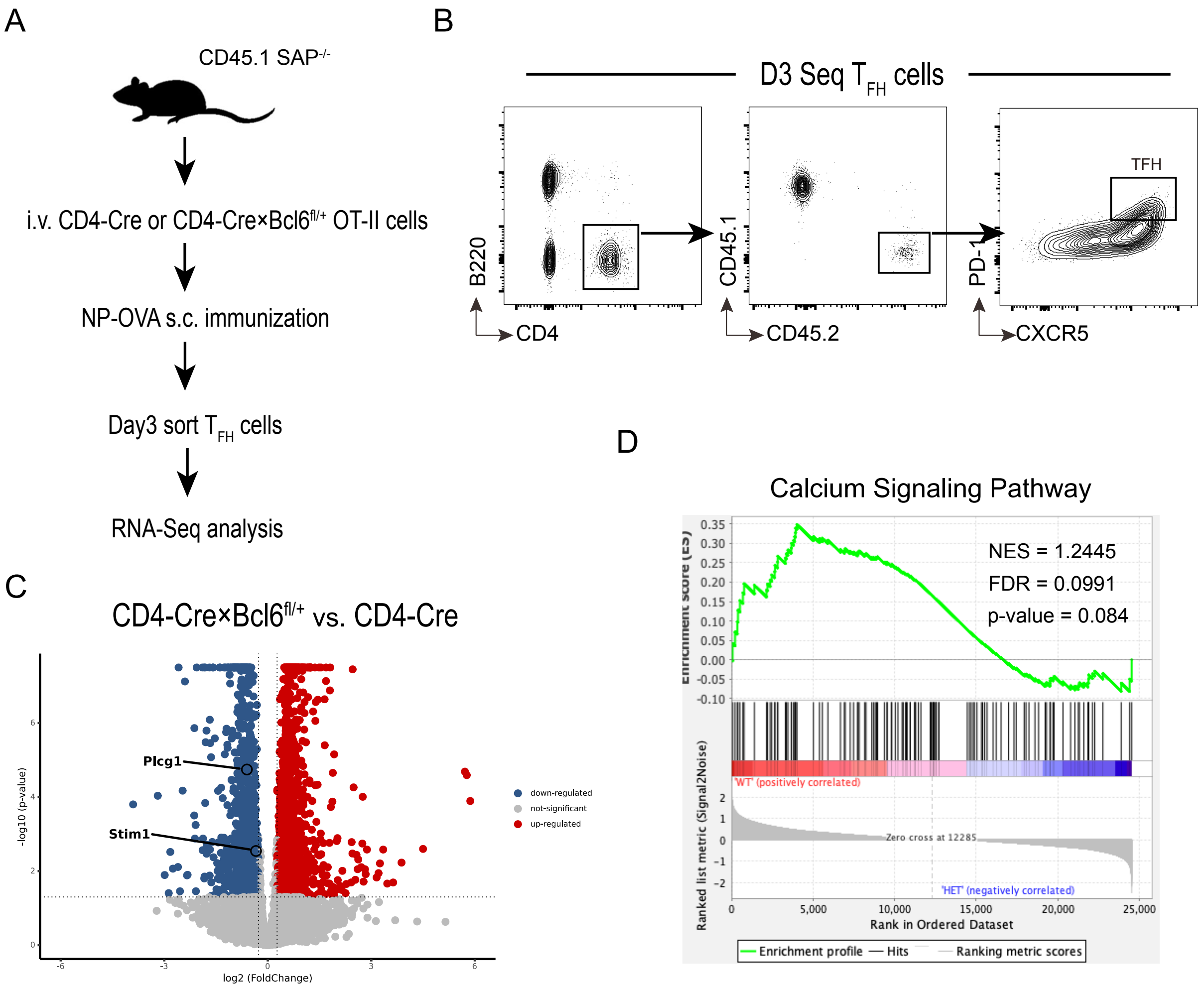

Figure S7. Reduced calcium signaling signature in BCL6-insufficient Tfh cells, related to Figure 3.

mRNA sequencing analysis of CD4-Crex*Bcl6*$^{fl/+}$ or CD4-Cre OT-II Tfh cells 3 days post immunization. **A,** The experimental scheme. **B,** The gating strategy for sorting Tfh cells. **C,** A volcano plot highlighting genes differentially expressed (P<0.05, fold change⩾1.2) between CD4-Crex*Bcl6*$^{fl/+}$ and CD4-Cre Tfh cells. Genes upregulated in CD4-Crex*Bcl6*$^{fl/+}$ Tfh cells are in red, and downregulated are shown in blue. Circles highlight *Stim1* and *Plcg1*. **D,** Enrichment analysis comparing CD4-Crex*Bcl6*$^{fl/+}$ ("HET") or CD4-Cre ("WT") OT-II T cells for genes in the calcium signaling pathway as defined in the KEGG database. NES, normalized enrichment score; FDR, false discovery rate.
