# Supplementary material for: BCL6 controls contact-dependent help delivery during follicular T-B cell interactions
Source: Immunity. 2021 Oct 12;54(10):2245–2255.e4. doi: 10.1016/j.immuni.2021.08.003 (PMC8528402; doi:10.1016/j.immuni.2021.08.003)
Supplement: Document S2. Article plus supplemental information [file mmc8.pdf]

Immunity

Article

BCL6 controls contact-dependent help delivery during follicular T-B cell interactions

Highlights

- Both copies of *Bcl6* gene are required for efficient follicular T-B cell entanglement
- BCL6 is required for calcium signaling in Tfh cells and CD40L delivery to B cells
- Expression of Tfh markers responds to acute perturbation of T-B cell interactions
- Defective functions of BCL6-insufficient T cells are rescued by CD40L overexpression

Authors

Dan Liu, Jiacong Yan, Jiahui Sun, ..., Ye Li, Xingxing Shao, Hai Qi

Correspondence

qihai@tsinghua.edu.cn

In brief

BCL6 is required for Tfh cell development. Liu et al. show that BCL6 controls calcium signaling of Tfh cells, follicular T-B cell entanglement, and efficient T cell-to-B cell CD40L delivery and promotes Tfh cell maintenance partly in a T cell non-autonomous manner.

Liu et al., 2021, Immunity *54*, 2245–2255
October 12, 2021 © 2021 The Author(s). Published by Elsevier Inc.
https://doi.org/10.1016/j.immuni.2021.08.003

CellPress

Immunity

CellPress
OPEN ACCESS

Article

BCL6 controls contact-dependent help delivery during follicular T-B cell interactions

Dan Liu,[1,2,3,7,8] Jiacong Yan,[1,2,3,4,7,9] Jiahui Sun,[1,2,3,7] Bo Liu,[1,2,3] Weiwei Ma,[2,3] Ye Li,[2,3] Xingxing Shao,[1,2,3,4] and Hai Qi[1,2,3,4,5,6,10,*]
[1]Tsinghua-Peking Center for Life Sciences, Tsinghua University, Beijing 100084, China
[2]Laboratory of Dynamic Immunobiology, Institute for Immunology, Tsinghua University, Beijing 100084, China
[3]Department of Basic Medical Sciences, School of Medicine, Tsinghua University, Beijing 100084, China
[4]School of Life Sciences, Tsinghua University, Beijing 100084, China
[5]Beijing Key Laboratory for Immunological Research on Chronic Diseases, Tsinghua University, Beijing 100084, China
[6]Beijing Frontier Research Center for Biological Structure, Tsinghua University, Beijing 100084, China
[7]These authors contributed equally
[8]Present address: Department of Microbiology and Immunology, University of California, San Francisco, San Francisco, CA
[9]Present address: Reproductive Medical Center, The First People's Hospital of Yunnan Province, Kunming, Yunnan, China
[10]Lead contact
*Correspondence: qihai@tsinghua.edu.cn
https://doi.org/10.1016/j.immuni.2021.08.003

SUMMARY

BCL6 is required for development of follicular T helper (Tfh) cells to support germinal center (GC) formation. However, it is not clear what unique functions programmed by BCL6 can explain its absolute essentiality in T cells for GC formation. We found that ablation of one *Bcl6* allele did not appreciably alter early T cell activation and follicular localization but inhibited GC formation and Tfh cell maintenance. BCL6 impinged on Tfh calcium signaling and also controlled Tfh entanglement with and CD40L delivery to B cells. Amounts of BCL6 protein and nominal frequencies of Tfh cells markedly changed within hours after strengths of T-B cell interactions were altered *in vivo*, while CD40L overexpression rectified both defective GC formation and Tfh cell maintenance because of the BCL6 haploinsufficiency. Our results reveal BCL6 functions in Tfh cells that are essential for GC formation and suggest that BCL6 helps maintain Tfh cell phenotypes in a T cell non-autonomous manner.

INTRODUCTION

The germinal center (GC) is the primary site in which high-affinity, long-lived plasma cells and memory B cells develop (Berek et al., 1991; Coico et al., 1983; Jacob et al., 1991; MacLennan, 1994). GC development requires activation of antigen-specific helper T cells and their provision of contact-dependent help signals such as the CD40 ligand (CD40L) to drive optimal clonal expansion, survival, and differentiation of antigen-specific B cells (Banchereau et al., 1994; Victora and Nussenzweig, 2012). While all helper T cells synthesize CD40L and can in principle promote B cell proliferation *in vitro* (Koguchi et al., 2012; Koguchi et al., 2007), follicular T helper (Tfh) cells, a subset of effector $CD4^+$ T cells localized in the follicle (Campbell et al., 2001; Schaerli et al., 2000), are specialized in promoting the GC development *in vivo* (Crotty, 2011; Qi, 2016; Vinuesa et al., 2016b).

Following antigen activation, the initial encounter between antigen-specific helper T cells and B cells takes place in the interfollicular region and at the border between the T cell zone and the follicle (Garside et al., 1998; Kerfoot et al., 2011). Here, these cells form mobile but stable contacts that can last for tens of minutes to hours, invariably with migratory B cells leading the conjugate pair (Okada et al., 2005; Qi et al., 2008). As antigen-specific B cells clonally expand on helper signals provided by T cells, they migrate back into the follicle in preparation for GC formation (Cyster, 2010). A fraction of activated helper T cells concomitantly migrate into the follicle, in a manner that is independent of the cognate B cell partner (Vinuesa and Cyster, 2011). Follicular localization of activated helper T cells depends on the chemokine receptor CXCR5 (Arnold et al., 2007; Haynes et al., 2007), which senses the follicle-derived directional cue CXCL13 (Förster et al., 1996; Gunn et al., 1998), and inducible costimulator molecule (ICOS), which promotes follicular T cell motility upon engagement by ICOS ligand (ICOSL) displayed on bystander B cells (Xu et al., 2013).

BCL6 is a transcription repressor that is required for the development and maintenance of Tfh cells and Tfh cell-supported GC formation (Johnston et al., 2009; Nurieva et al., 2009; Yu et al., 2009). However, little is known about biological processes that are both controlled by BCL6 and absolutely essential for GC formation. Chromatin-binding and transcriptomic analyses have revealed many genes that are potentially subject to BCL6-mediated suppression (Hatzi et al., 2015; Liu et al., 2016b). These include signaling molecules and transcription factors that promote terminal effector T cell differentiation and chemotactic receptors that are incompatible with follicle-directed movement

This is an open access article under the CC BY license (http://creativecommons.org/licenses/by/4.0/).

and localization. $CD4^+$ T cells genetically deficient in BCL6 cannot normally express CXCR5 and do not relocalize into the follicle. Although the transcription factor ASCL2 instead of BCL6 directly binds to and regulates *Cxcr5* gene expression (Hatzi et al., 2015; Liu et al., 2014; Liu et al., 2016b), BCL6 can suppress the expression of the transcription factor Id2 and thus release Id2-mediated suppression of E proteins, which can positively drive CXCR5 expression (Liu et al., 2014; Shaw et al., 2016). These results that indicate follicular localization is a key aspect for which BCL6 is required in order to promote Tfh cell development and function (Qi, 2016). However, whereas CXCR5 ablation or prevention of CCR7 downregulation in T cells causes a defect in follicular recruitment, GCs can still form inside the follicle with the help of CXCR5 null Tfh cells and suffer a relatively mild 2-fold reduction in magnitude (Arnold et al., 2007; Haynes et al., 2007). This is in sharp contrast to the outcome of T cell-specific *Bcl6* ablation, which completely abrogates GC formation (Johnston et al., 2009; Nurieva et al., 2009; Yu et al., 2009). Therefore, beyond follicular recruitment, BCL6 must regulate additional aspects of T cell biology that are uniquely important for helping B cells (Qi et al., 2014). One unique aspect of T cell help for GC formation is its dependence on physical cell-cell contact, which takes on highly dynamic forms *in vivo* (Liu et al., 2015; Okada et al., 2005; Qi et al., 2008; Shulman et al., 2014). Such contacts require active signaling through the T cell antigen receptor (TCR), which is further enhanced by accessory molecules such as SAP and ICOS (Cannons et al., 2010; Chu et al., 2014; Liu et al., 2015; Qi et al., 2008). It has been found that, when deprived of B cell-mediated antigen presentation for several days, frequencies of Tfh cells would decrease, even though no unique instructional signals from B cells, other than antigen presentation, are required for maintaining these Tfh cells (Baumjohann et al., 2013; Deenick et al., 2010). The simplest explanation is that, in a cell-autonomous manner, BCL6 instructs a stable program of Tfh cell differentiation, albeit requiring periodic TCR stimulation to maintain, polarize, and/or manifest (Crotty, 2014; Vinuesa et al., 2016a).

In this study, we combined static and intravital imaging analyses to investigate how BCL6 regulates cell biology of Tfh cells and found that BCL6 impinges on calcium signaling of T cells, regulates dynamics of T-B cell interactions and efficiency of help delivery to B cells, and promotes Tfh cell maintenance partly in a T cell non-autonomous manner.

RESULTS

BCL6 is haploinsufficient for recruitment-independent helper functions

Antigen-activated BCL6-deficient T cells exhibit an intrinsic defect in follicular localization, as demonstrated in mixed bone-marrow chimera (Yu et al., 2009), presumably because of their failure to properly shift the chemo-sensing preference by upregulating CXCR5 and downregulating CCR7 (Johnston et al., 2009; Nurieva et al., 2009; Yu et al., 2009). Lack of follicular localization of BCL6-deficient T cells precludes analysis of potentially important functions for BCL6 in the follicular phase, which appears likely given the phenotypic discrepancy in GC formation between T cell-intrinsic BCL6 deficiency and CXCR5 deficiency. To circumvent this problem, we first tested T cells isolated from heterozygous $Bcl6^{+/-}$ mice. As shown in Figure 1A, $Bcl6^{+/-}$ and $Bcl6^{+/+}$ OT-II T cells expressed grossly similar amounts of CXCR5 and were recruited into the follicle with comparable efficiencies 3–4 days after NP-OVA immunization (Figures 1B and 1C), as measured by the ratio between the follicular and the T zone densities of donor OT-II T cells (the follicle recruitment index, FRI; Figure S1). These data indicate that one intact allele of the *Bcl6* gene is sufficient for ensuring follicular recruitment of helper T cells. Importantly, however, $Bcl6^{+/-}$ OT-II T cells remained significantly less competent in promoting GC formation (Figure 1D) or maintaining the associated $CXCR5^+$ Tfh cell phenotype over a longer term, a defect that manifested 8 days after immunization (Figure 1E). Therefore, both alleles of the *Bcl6* gene must be expressed to normally orchestrate helper functions inside the follicle to maintain Tfh cell phenotype and support GC formation.

BCL6 is not required for maintaining stable T-B cell interactions at the T zone-follicle border

Stable T-B cell interactions, in the form of long-lasting conjugate pairs at the T zone-follicle border (Okada et al., 2005; Qi et al., 2008) or entangled couples inside the follicle (Liu et al., 2015; Shulman et al., 2014), are required for GC formation and maintenance. To explore whether BCL6 would impinge on cognate T-B cell interactions *in vivo*, we first visualized $Bcl6^{+/-}$ and $Bcl6^{+/+}$ OT-II T cells interacting with MD4 B cells 36–48 h after HEL-OVA immunization by 2-photon intravital microscopy. As previously reported, this is a time point when activated T cells form long-lasting conjugate pairs with cognate B cells at the T zone-follicle border (Okada et al., 2005; Qi et al., 2008). We found that both $Bcl6^{+/-}$ and $Bcl6^{+/+}$ OT-II T cells formed comparably stable conjugates with MD4 B cells (Figure S2; Video S1 and S2). Therefore, in the early phase of the immune response at the T zone-follicle border, one intact *Bcl6* allele is sufficient for activated T cells to maintain long-lasting contacts with cognate B cells.

BCL6 is required for T cells to maintain entangled contacts with cognate B cells

As the response progresses into the follicular stage, antigen-specific T-B cell interactions become shortened in duration but extensive in area of contact, frequently assuming the entangled configuration (Liu et al., 2015). To determine whether one intact *Bcl6* allele is still sufficient for maintaining entangled contacts with antigen-specific B cells inside the follicle, CFP-expressing control $Bcl6^{+/+}$ OT-II cells and dsRed-expressing $Bcl6^{+/-}$ or $Bcl6^{+/+}$ OT-II cells were co-transferred together with GFP-expressing MD4 B cells into B6 recipients that were subsequently immunized with HEL-OVA. We conducted intravital imaging 96 h post immunization, when large numbers of OT-II T cells and MD4 B cells could be seen in the follicle. As shown in Videos S3 and S4 and Figures 2A–2C, contacts between $Bcl6^{+/-}$ OT-II T cells and MD4 B cells were comparable in duration to those between $Bcl6^{+/+}$ OT-II T cells and the same cohort of MD4 B cells; however, the normalized surface area of contacts, or the extent of entanglement measured by the surface engagement index (SEI) as previously defined (Liu et al., 2015) (Figure S3), was significantly reduced for $Bcl6^{+/-}$ OT-II T cells, revealing an haploinsufficient role for BCL6 in maintaining efficient T-B cell entanglement.

CellPress
OPEN ACCESS

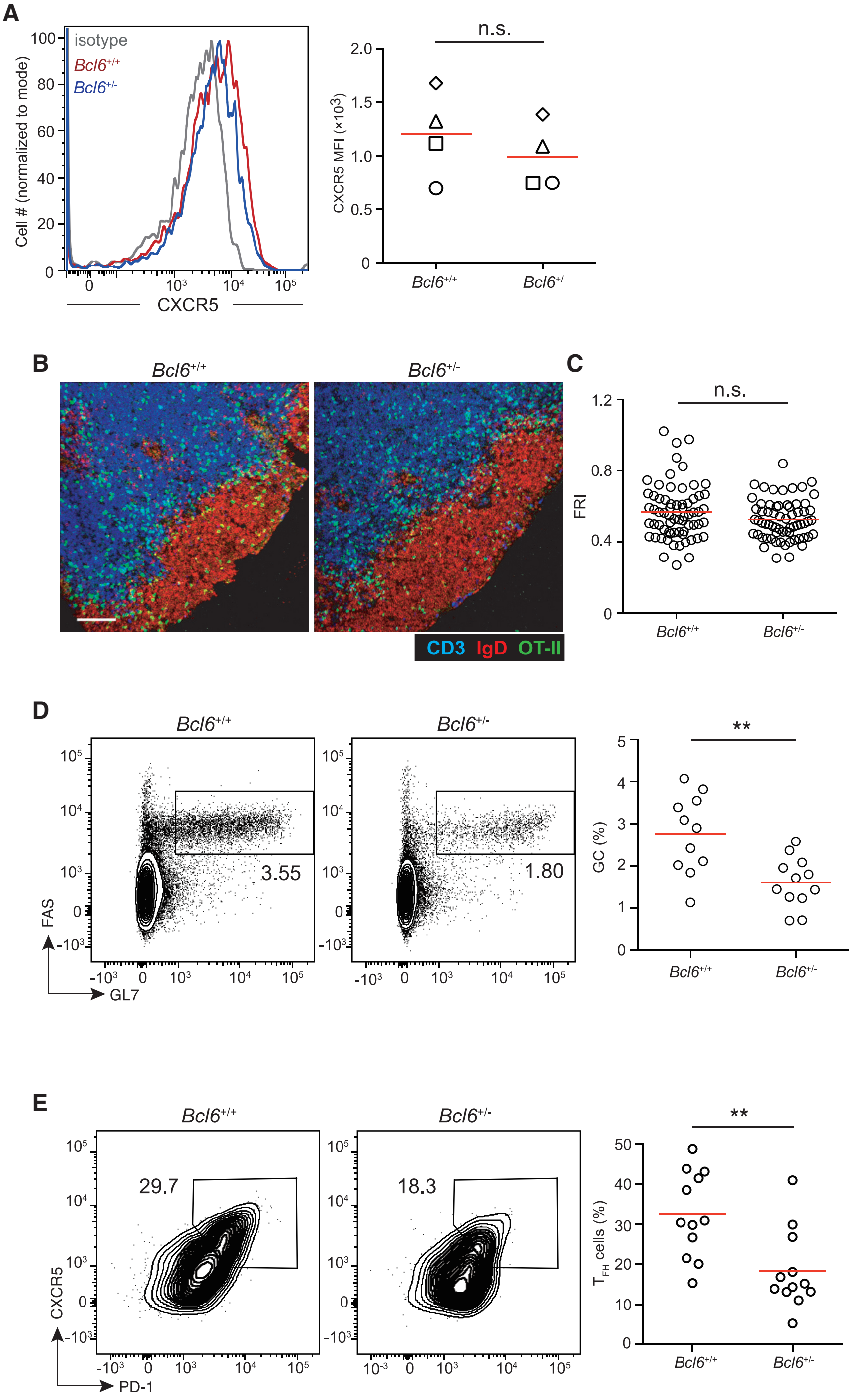

Figure 1. Haploinsufficiency of BCL6 impairs the follicular response

(A) Representative histograms and MFIs of surface CXCR5 expression on OT-II T cells of indicated genotypes in draining lymph nodes 4 days after NP-OVA immunization. Each symbol represents one experiment in which cells from 2–3 mice of each group were pooled. n.s., not significant.

(B and C) Representative distribution patterns (B) and FRIs (C) of GFP-transduced *Bcl6*$^{+/+}$ and *Bcl6*$^{+/-}$ OT-II T cells in draining lymph nodes as in (A). Each symbol represents one follicle and its adjacent T cell zone, and lines denote means. One of 2 experiments with similar results is shown, and each group had 3 mice per experiment. n.s., not significant. Scale bar, 100 μm.

(D and E) Representative flow-cytometry profiles and frequencies of FAS^{hi} GL7^{hi} GC B cells in total CD19^{+} B cells (D) or flow-cytometry profiles and frequencies of CXCR5^{hi} PD1^{hi} Tfh cells in OT-II T cells (E) 8 days after NP-OVA immunization in *Sap*$^{-/-}$ mice that received OT-II T cells of indicated genotypes. Each symbol represents one mouse, and lines denote the means. Data are pooled from 3 independent experiments, with at least three mice per group per experiment. **p < 0.01.

See also Figure S1.

T-B cell entanglement is associated with heightened calcium signaling in T cells (Liu et al., 2015). We thus conducted calcium imaging of *Bcl6*$^{+/-}$ or *Bcl6*$^{+/+}$ OT-II T cells interacting with MD4 B cells in the follicle after HEL-OVA immunization, using a fluorescence resonance energy transfer (FRET)-based calcium reporter YC-Nano-50^{CD} (Horikawa et al., 2010), as previously used in Liu et al. (2015). As exemplified in Figure 3A and Videos S5 and S6, contacts between *Bcl6*$^{+/+}$ OT-II T cells and MD4 B cells were more frequently associated with pronounced calcium signaling than those between *Bcl6*$^{+/-}$ T cells and MD4 B cells. The fractional change in the normalized FRET signal intensity, $\Delta R_t/R_0$, reflects the change in intracellular (Cheli et al., 2016) at time *t* as compared with the no-contact reference state *0* of the same T cell prior to a contact. The mean $\Delta R_t/R_0$ measures the average calcium signaling intensity of a T cell in contact with a B cell. Quantitatively, *Bcl6*$^{+/-}$ OT-II T cells exhibited significantly lower mean $\Delta R_t/R_0$ values than control T cells (Figure 3B; mean ± SEM of mean $\Delta R_t/R_0$ values for 65 *Bcl6*$^{+/+}$ versus 64 *Bcl6*$^{+/-}$ contacts: 29% ± 6% versus 8% ± 4%, p < 0.01). To gauge the overall productivity of contact events involving the two T cell types, we used the time-integrated calcium-response index (TICRI) as previously described (Liu et al., 2015), which is calculated by multiplying mean $\Delta R_t/R_0$ of each contact with its duration. As shown in Figure 3C, TICRIs of *Bcl6*$^{+/-}$ cells were lower by ~3-fold (*Bcl6*$^{+/+}$: 134 ± 34; *Bcl6*$^{+/-}$: 28 ± 13; p < 0.01). Combined together, these data reveal a haploinsufficient role for BCL6 in promoting optimal calcium signaling and T-B cell entanglement inside the follicle.

The absence of BCL6 impairs CD40L externalization

During T-B cell entanglement in the follicle or GCs, calcium-signaling-mediated CD40L externalization is a prerequisite for

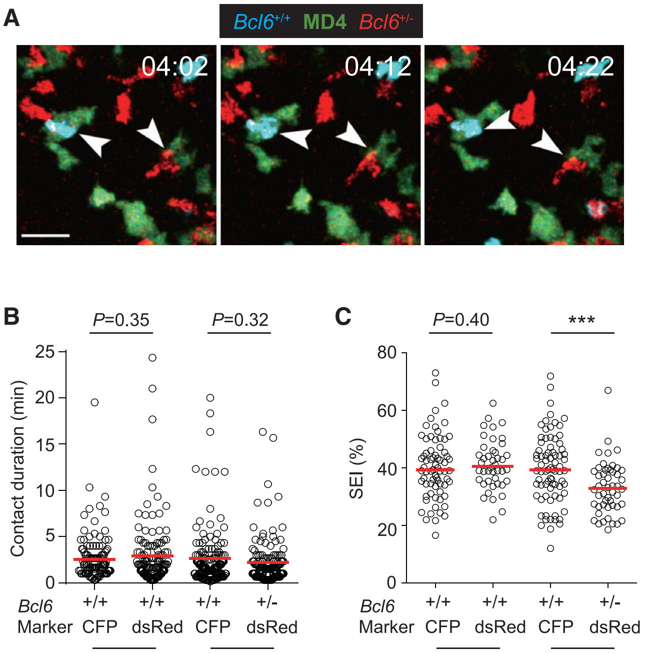

Figure 2. BCL6 is required for efficient T-B cell entanglement inside the follicle

(A–C) GFP-expressing MD4 B cells, control CFP-expressing *Bcl6*$^{+/+}$ and dsRed-expressing test *Bcl6*$^{+/-}$, or test *Bcl6*$^{+/+}$ OT-II T cells were visualized 4 days after immunization. (A) *Bcl6*$^{+/+}$ and *Bcl6*$^{+/-}$ OT-II T cells interacting with MD4 B cells, with contacts highlighted by arrowheads. Scale bar, 20 μm. See corresponding Video S4. Duration (B) and SEI (C) of contacts of indicated types. See corresponding Videos S3 and S4. Each symbol is one contact, and lines denote the means. Data are pooled from three independent experiments. ***$p < 0.001$.
See also Figure S3.

contact-dependent CD40L delivery from Tfh cells to cognate B cells (Liu et al., 2015). Blockade of CD40L signaling to antigen-activated B cells already in the follicular phase (e.g., as late as 50–60 h in the model of MD4 and OT-II co-transfer) markedly impaired GC formation (Figure S4). While surface CD40L expression was comparable between *Bcl6*$^{+/-}$ than *Bcl6*$^{+/+}$ Tfh cells *in vivo*, the total amount of CD40L protein detected by intracellular staining was lower in *Bcl6*$^{+/-}$ cells, consistent with impaired calcium signaling in T cells when BCL6 expression is compromised (Figures 4A and 4B). Importantly, when T cells were stimulated via anti-CD3 to mobilize CD40L from the intracellular store onto the plasma membrane over a brief period of 15 min, a calcium-signaling-dependent process, *Bcl6*$^{+/-}$ Tfh cells mobilized significantly less amounts of CD40L (Figures 4C and 4D). These data suggest that, despite being able to express CD40L, BCL6-insufficient T cells could not efficiently deliver CD40L during those brief contacts with cognate B cells, most likely as a result of reduced calcium-signaling efficiency.

BCL6 insufficiency impairs CD40L delivery to B cells in a polyclonal response

Our results have so far revealed two major defects of BCL6-insufficient T cells inside the follicle that can be explained by an impairment of TCR-triggered calcium flux: reduced T-B cell physical contact and reduced CD40L externalization. These helper defects can readily explain impairment of B cell expansion and GC formation. On the other hand, it is difficult to explain why *Bcl6*$^{+/-}$ T cells exhibited grossly normal Tfh cell development at the beginning but could not maintain their Tfh cell phenotype over time. In particular, the effect of missing one copy of *Bcl6* gene in T cells appeared to have a more immediate and pronounced repercussion on B cells being helped rather than on the helper cells per se.

Our use of OT-II and MD4 receptor-transgenic cells, while necessary for intravital imaging experiments to examine cell-cell interaction dynamics, might reveal functional defects that do not normally manifest. Moreover, germline *Bcl6* ablation might have unintended consequences. To ameliorate these concerns, we bred *Cd4-cre* and *Bcl6*$^{fl/fl}$ mice to create T cell-specific BCL6-deficient animals. In replicating our observations with the heterozygous germline *Bcl6*$^{+/-}$ (Figure 1), we found that *Cd4-cre* × *Bcl6*$^{fl/+}$ OT-II T cells were able to express similar amounts of CXCR5 following 4 days of activation *in vivo* but were significantly less competent in promoting GC formation or maintaining the CXCR5^{+} phenotype by day 8 (Figure S5). Next, we transferred splenocytes from *Cd4-cre*, *Cd4-cre* × *Bcl6*$^{fl/+}$, or *Cd4-cre* × *Bcl6*$^{fl/fl}$ mice into sub-lethally irradiated CD45.1 SAP-deficient mice and immunized these recipients with NP-KLH (Figure 5A). In this system, normal polyclonal B cells would respond to NP-KLH with cognate T cell help provided by exogenous wild-type (*Cd4-cre*), BCL6-insufficient (*Cd4-cre* × *Bcl6*$^{fl/+}$), or BCL6-deficient (*Cd4-cre* × *Bcl6*$^{fl/fl}$) T cells from a polyclonal repertoire. The endogenous T cells cannot support GC formation because of the SAP deficiency (Qi et al., 2008). By day 13, when the NP-KLH-induced GC response typically peaked in wild-type mice, the group with BCL6-deficient T cells had far fewer T cells exhibiting the Tfh phenotype and almost no detectable GCs, as expected; with BCL6-insufficient T cells as helper T cells, the magnitude of the GC response was halved, while the frequency of T cells exhibiting the Tfh phenotype was barely reduced (Figures 5B and 5C). These data indicate that, in a polyclonal response to immunization, both copies of the *Bcl6* gene are required for normal helper T cell activities to support GC formation, which is, again, more sensitive to the BCL6 insufficiency in T cells than is T cell development into Tfh cells per se (appearance of CXCR5^{+}PD-1^{+} T cells).

Next, to look for signs that BCL6-insufficient T cells may not efficiently deliver CD40L to B cells in a polyclonal response *in vivo*, we transferred wild-type (*Cd4-cre*) or BCL6-insufficient (*Cd4-cre* × *Bcl6*$^{fl/+}$) OT-II T cells into SAP-deficient mice and immunized these mice with NP-OVA (Figure S6A). By day 3 post immunization, we sort-purified NP-binding B cells that were developing into GCs (Figure S6B) and conducted RNA sequencing (RNA-seq) analyses of their transcriptome. Using a gene expression signature induced by CD40 signaling in B cells (Basso et al., 2004) for enrichment analysis, we found such CD40-induced gene set expression was significantly deprived in those NP-binding B cells helped by *Cd4-cre* × *Bcl6*$^{fl/+}$ T cells (Figure S6C). These data suggest BCL6 insufficiency in T cells leads to impaired CD40L signaling to B cells.

BCL6 and other Tfh cell markers depend on acute T-B cell interactions

It has been reported that, when Tfh cells are deprived of reciprocal B cell stimulation for several days, Tfh cell maintenance

CellPress
OPEN ACCESS

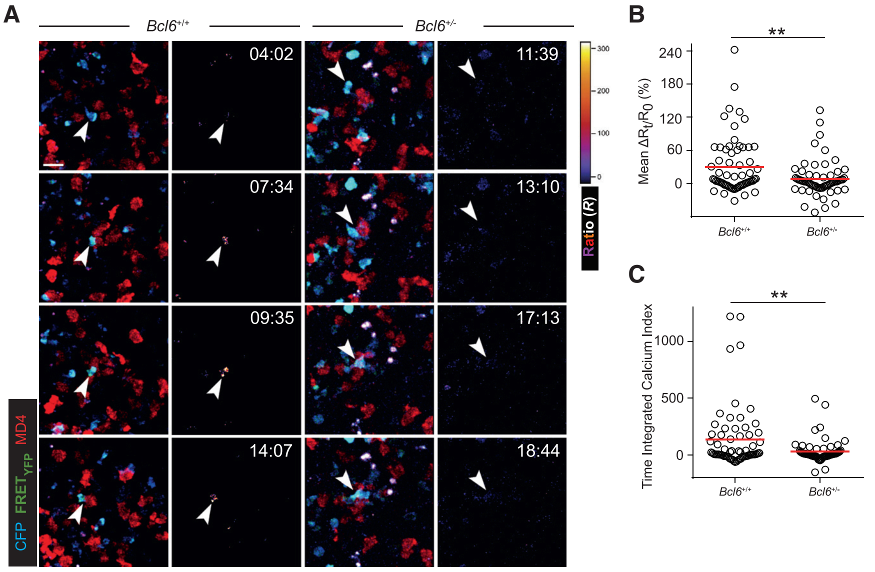

Figure 3. BCL6 is required for optimal calcium signaling during T-B cell entanglement *in vivo*

(A–C) dsRed-expressing MD4 B cells and YC-Nano-50^{CD}-transduced *Bcl6*$^{+/+}$ or *Bcl6*$^{+/-}$ OT-II T cells were visualized in B6 recipients 4 days after immunization. (A) Image sequences showing a *Bcl6*$^{+/+}$ and a *Bcl6*$^{+/-}$ OT-II T cell interacting with MD4 B cells. CFP fluorescence from the YC-Nano-50^{CD} reporter identifies T cells in the fluorescence overlays (columns 1 and 3), and ratiometric FRET signals are presented in the heatmap images (columns 2 and 4). Scale bar, 20 μm. See corresponding Videos S5 and S6. (B and C) Mean $\Delta R_t/R_0$ (B) and time-integrated calcium index (C) of individual contacts between *Bcl6*$^{+/+}$ (n = 65) or *Bcl6*$^{+/-}$ (n = 64) OT-II T cells and MD4 B cells. Lines denote the means. Data are pooled from four experiments. **p < 0.01.

is severely impaired (Baumjohann et al., 2013; Deenick et al., 2010). Based on a model of BCL6-instructed Tfh cell differentiation and maintenance (Crotty, 2014; Vinuesa et al., 2016a), one interpretation is that BCL6 may initiate an epigenetic Tfh cell program (Hatzi et al., 2015; Kroenke et al., 2012; Li et al., 2018; Liu et al., 2016a; Liu et al., 2012; Lu et al., 2011) that is inheritable but requires intermittent antigen stimulation to enforce over a long period of time. Alternatively, because markers of Tfh cells (namely CXCR5, PD-1, and BCL6) can be rapidly upregulated upon antigen stimulation *in vivo*, at least for naive T cells (Chen

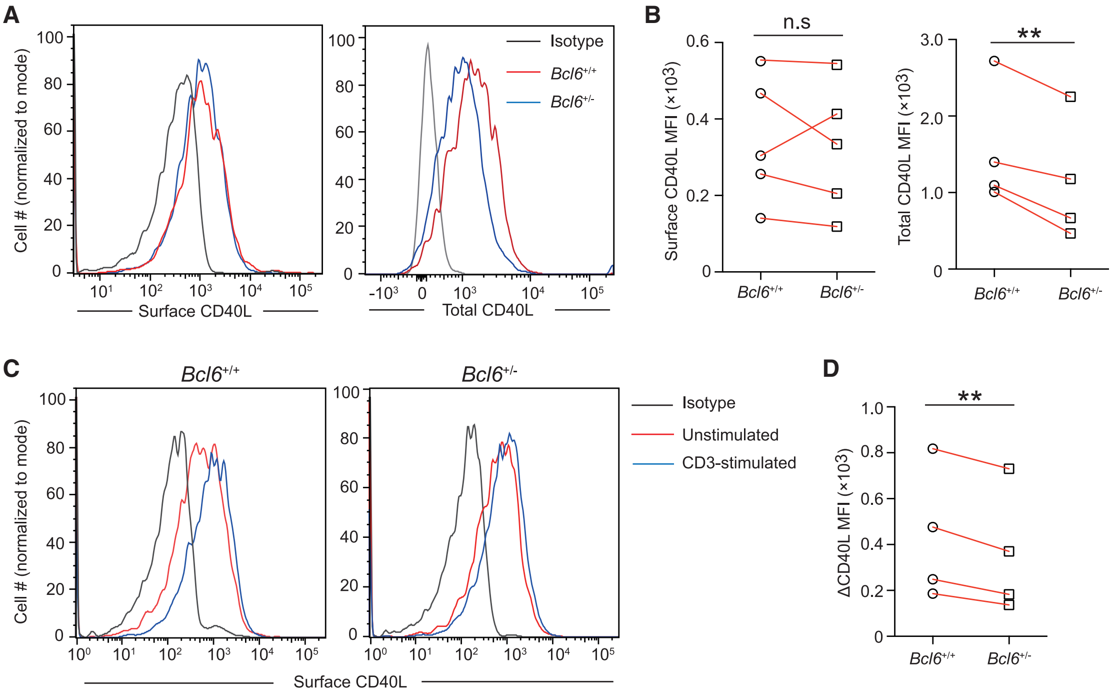

Figure 4. BCL6 is required for efficient CD40L delivery

OT-II T cells of indicated genotypes, 7–8 days after being activated *in vivo* in adoptive B6 hosts immunized with NP-OVA, were subjected to the CD40L mobilization assay as described in the STAR Methods.

(A) Representative histograms of cell surface CD40L (left) or total CD40L measured by intracellular staining (right) of *Bcl6*$^{+/+}$ and *Bcl6*$^{+/-}$ OT-II T cells.

(B) MFIs of CD40L staining for conditions in (A); each line represents one independent experiment for each comparison setup.

(C and D) CD40L mobilization by anti-CD3 in *Bcl6*$^{+/+}$ and *Bcl6*$^{+/-}$ OT-II T cells; representative histogram overlays (C) and changes in surface CD40L MFI (ΔMFI) following indicated stimulation (D). Each line represents one independent experiment. n.s. not significant, **p < 0.01.

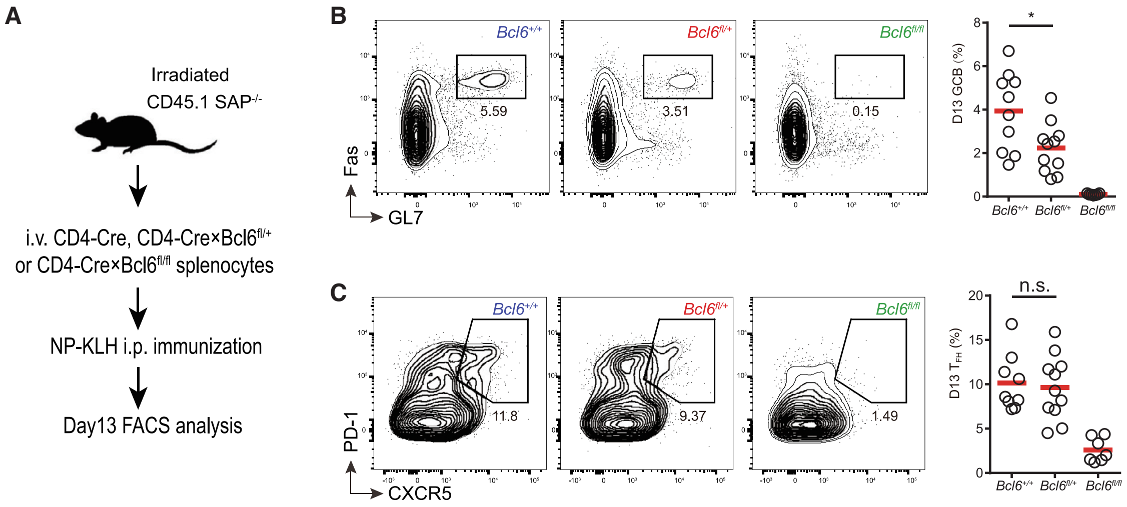

Figure 5. Haploinsufficiency of BCL6 impairs a polyclonal response

(A) The experimental scheme.

(B and C) Representative flow-cytometry profiles and frequencies of FAS^{hi} GL7^{hi} GC B cells in total B220^{+} B cells (B) or CXCR5^{hi}PD-1^{hi} Tfh cells in CD44^{hi} CD4 T cells of the donor origin (C) 13 days after NP-KLH immunization in CD45.1 *Sap*$^{-/-}$ recipients. All donors are on the CD4-Cre background, and genotypes of the Bcl6 allele are indicated. Each symbol represents one mouse, and lines denote the means. Data are pooled from 2 independent experiments. n.s., not significant. *$p < 0.05$.

et al., 2015; Kerfoot et al., 2011), it is also plausible that, even in the follicular phase of the response, reduced T-B cell interactions have a rapid repercussion on expression of markers of Tfh cells within a short time frame. Either of these two models could explain the inability of *Bcl6*$^{+/-}$ T cells to maintain Tfh cell phenotype in the follicular phase. A key distinction, however, is the timescale by which manipulation of follicular T-B cell interactions can affect the Tfh cell phenotype.

To test whether BCL6 and other markers of Tfh cells can be rapidly up- or downregulated upon acute manipulation of follicular T-B cell interactions, we took two approaches. First, we injected αDEC205 antibody linked to the ovalbumin-derived peptide epitope OVA$_{323-339}$ (αDEC-OVA) 100 h post HEL-OVA immunization of B6 mice that received OT-II T cells and MD4 B cells. Because as antigen-activated B cells upregulate DEC-205 as they develop into GC B cells, the αDEC-OVA treatment preferentially delivers OVA epitope to those B cells (Victora et al., 2010). Merely 15 h after αDEC-OVA injection, OT-II T cells in the spleen markedly upregulated CXCR5, PD-1, and BCL6 (Figure 6A), leading to doubling of the frequency of CXCR5^{hi}PD-1^{hi} cells (Figure 6B) without changing the overall OT-II abundance. BCL6 expression in cells already gated as CXCR5^{hi}PD-1^{hi} were also markedly increased by ∼3-fold after αDEC-OVA injection (Figure 6C). Second, we injected αCD40L antibody to block CD40-CD40L interactions between follicular OT-II and MD4 B cells. Within the same 15 h period, amounts of BCL6, CXCR5, and PD-1 decreased on OT-II T cells (Figure 6A), with their nominal Tfh cell frequency being reduced by approximately one-third (Figure 6B). Those CXCR5^{hi}PD-1^{hi} cells also showed a significant decrease in BCL6 expression (Figure 6C). These results suggest any BCL6-instructed, inheritable program that specifies and maintains the CXCR5^{hi}PD-1^{hi}BCL6^{+} Tfh cell phenotype would not be stable for longer than 15 h without exogenous input (e.g., B cell-mediated antigen presentation). Given how brief this 15 h period, it is plausible that BCL6 also helps maintain the CXCR5^{hi}PD-1^{hi} phenotype in a T cell non-autonomous manner by simply promoting cognate T-B cell interactions (Figures 3 and 4).

Defects in GC formation and Tfh cell maintenance associated with BCL6-compromised T cells can be rescued

If there is a T cell non-autonomous mechanism of BCL6-dependent Tfh cell maintenance as deduced above, one would predict that if CD40L is made more abundantly available in BCL6-insufficient T cells to compensate for the impaired delivery of CD40L, both the failure of normal GC formation and Tfh cell maintenance would be rescued. On the other hand, if BCL6 only instructs and maintains a Tfh cell fate in a strictly T cell autonomous manner, CD40L overexpression should not rescue the Tfh cell defect of BCL6-insufficient cells, even if it might rescue defective GC formation. To test this, we overexpressed CD40L in *Bcl6*$^{+/-}$ and *Bcl6*$^{+/+}$ OT-II T cells by retroviral transduction, transferred these cells together with MD4 B cells into SAP-deficient mice, and examined MD4 GC formation and OT-II Tfh cell development. As shown in Figure 7A, while control-transduced *Bcl6*$^{+/-}$ OT-II T cells were not able to support normal GC formation, *Bcl6*$^{+/-}$ OT-II T cells overexpressing CD40L now supported a similar magnitude of GC formation as compared with the *Bcl6*$^{+/+}$ counterpart. Importantly, the reduced CXCR5^{hi}PD1^{hi} Tfh cell frequency of *Bcl6*$^{+/-}$ cells was rectified to a comparable magnitude of control-transduced *Bcl6*$^{+/+}$ cells (Figure 7B). Taken together, these data suggest that, in the follicular phase, the key biological process that BCL6 controls is contact-dependent help delivery to B cells, which is not only essential for GC formation but also for Tfh cell maintenance in a T cell non-autonomous manner.

CellPress
OPEN ACCESS

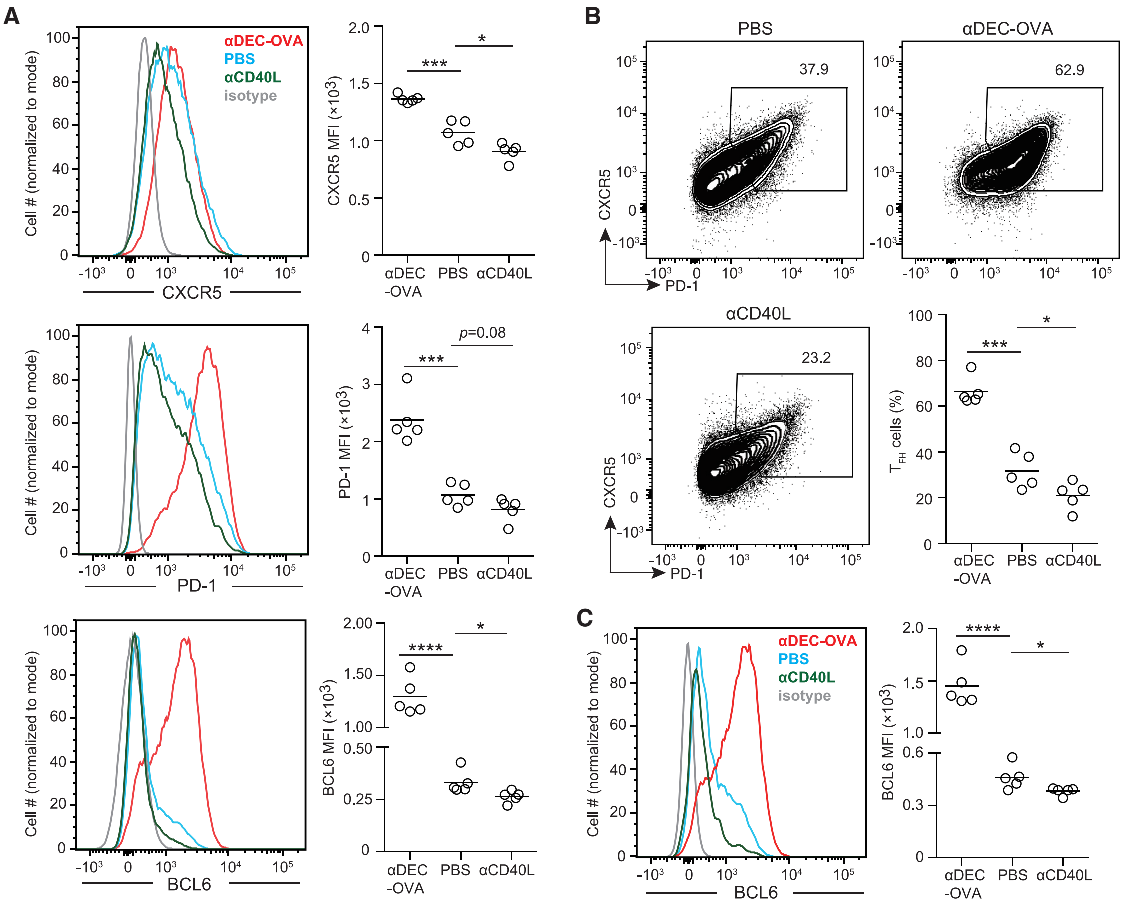

Figure 6. Maintenance of BCL6 and other Tfh cell markers requires constant T-B cell interactions

(A–C) CXCR5, PD-1, and BCL6 expression after acute antigen stimulation and T-B cell cooperation blockade *in vivo*. (A) Histograms and MFI statistics of indicated markers on transferred OT-II T cells 15 h after PBS, αDEC-OVA, or αCD40L injection. (B) Frequencies of OT-II Tfh cells gated as CXCR5^{hi} PD1^{hi} cells. (C) BCL6 expression by CXCR5^{hi} PD1^{hi} OT-II Tfh cells as gated in (B). Each symbol in scatterplots denotes one mouse. One of four experiments with similar results is shown. *p < 0.05, ***p < 0.001, ****p < 0.0001.

BCL6 is required for normal expression of calcium-signaling-related genes

Finally, in trying to understand how BCL6 may regulate calcium signaling in Tfh cells, which is important for T-B cell entanglement and for CD40L delivery, we noted that regulatory regions of human *STIM1* gene are bound by BCL6 in human Tfh cells (Hatzi et al., 2015). We conducted mRNA sequencing of BCL6-sufficient (*Cd4-cre*) and BCL6-insufficient (*Cd4-cre* × *Bcl6*$^{fl/+}$) OT-II T cells sort-purified 3 days after activation *in vivo* by NP-OVA immunization. As shown in Figure S7, when one copy of the *Bcl6* gene was missing, CXCR5^{+}PD-1^{+} Tfh cells differentially expressed many genes; among those downregulated were *Stim1* and *Plcg1* (which code for STIM1 and PLCγ1, respectively) that both impinge on calcium signaling downstream of TCR activation. Moreover, KEGG pathway analyses revealed that the calcium-signaling-related pathway was generally upregulated in wild-type as compared with BCL6-insufficient Tfh cells (Figure S7). Together, these data support the notion that BCL6 controls follicular T-B cell interactions by regulating multiple target genes involved in antigen-triggered calcium signaling in T cells.

DISCUSSION

Our study reveals a previously unappreciated role for BCL6 in enhancing antigen-triggered calcium signaling in Tfh cells and promoting their entangled interactions with and CD40L delivery to cognate B cells, all specifically in the follicular phase of a primary response. Combined with its obligate role in regulating CXCR5 expression and associated follicular recruitment (Liu et al., 2014; Shaw et al., 2016), our findings provide a more complete picture for biological processes that BCL6 actually controls in T cells to render its essentiality for GC formation. At least in the model of protein immunization used here, early CXCR5 upregulation and Tfh cell development proceeded normally as far as one copy of *Bcl6* allele is intact, whereas follicular functions of BCL6 in T cells required both alleles intact. Given the fact that BCL6 is very abundantly expressed in normal Tfh cells, we speculate those target genes involved in BCL6-dependent calcium signaling might utilize low-affinity binding sites such that only a high concentration of nuclear BCL6, achievable with expression from both alleles, can ensure productive engagement and regulation.

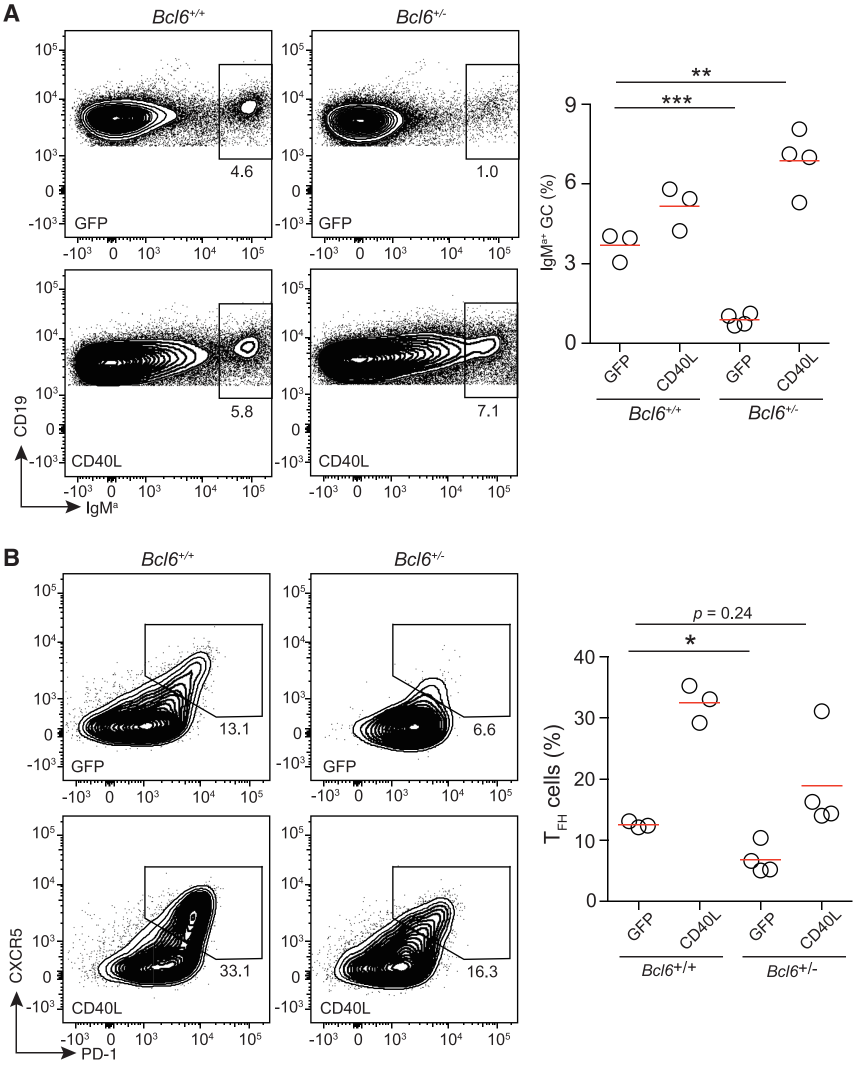

Figure 7. Rescue of BCL6-insufficient Tfh cells secondary to GC rectification

(A) Representative flow-cytometry profiles (left) and frequencies of IgM^{a+} MD4 GC B cells in total $CD19^+$ B cells (right) 5 days after HEL-OVA immunization of *Sap*$^{-/-}$ mice that received transfer of MD4 B cells and *Bcl6*$^{+/+}$ or *Bcl6*$^{+/-}$ OT-II T cells transduced with a control GFP or CD40L-expressing vector. Each symbol in the scatterplot (right) represents one mouse, and lines denote the means. One of three independent experiments with similar results is shown. **$p < 0.01$, ***$p < 0.001$.

(B) Representative flow-cytometry profiles (left) and frequencies of $CXCR5^{hi}$ $PD1^{hi}$ Tfh cells in OT-II T cells as in (A). Each symbol shape represents one mouse, and lines denote the means. One of three independent experiments with similar results is shown. *$p < 0.05$.

Although the capacity to produce CD40L is not unique to the Tfh cell subset, by definition only Tfh cells are tasked to deliver CD40L to B cells inside the follicle. CD40L delivery requires cell-cell contacts of sufficient stability, strength, and quality. The follicular microenvironment is highly dynamic, with all cells being in constant and fast migration, increasing the threshold for any T-B cell contacts to be functionally productive. This is probably the main reason why two copies of the *Bcl6* gene are not required for stable T-B cell interactions at the T zone-follicle border but become essential for those entangled contacts inside the follicle.

Expression of BCL6, CXCR5, and PD-1—markers universally used to identify Tfh cells and to assess the underlying developmental program—could markedly change within 15 h, depending the status of follicular T-B cell interactions *in vivo*. In additional work, αDEC-OVA was found to drive such changes within 7 h (unpublished data). Our results set the upper time limit by which manifestation of a Tfh cell program can be maintained in the follicular phase of a primary response following protein immunization. These results are also consistent with the fact that none of Tfh cell-associated genes are known to be epigenetically rendered open or shut across multiple mitoses in single Tfh cells by a BCL6-mediated mechanism, even though transcription of Tfh cell-associated genes may depend on BCL6, directly or indirectly.

It can be argued that because only three main markers of Tfh cells are examined in the current study, other Tfh cell-associated molecules or pathways, such as those underlying BCL6 follicular functions reported here, might be programmed in a more long-lasting mode. This possibility warrants further investigation. However, our results do caution interpreting changes in CXCR5, PD-1, and BCL6 markers, either in terms of expression intensities or frequencies of marker-positive cells (universally used to report Tfh cell frequencies in the literature), as demonstration of differentiation, trans-differentiation, or plasticity events being induced by any experimental perturbation—particularly when the perturbation is applied days or even weeks before reading the outcome. Such perturbation could be simply changing the strength of TCR stimulation and T-B cell interactions hours before the assay readout. At the same time, we should also note the fact that T cell CXCR5 expression, besides its extreme flexibility that is conditional upon T-B cell interactions in the follicle, might be subjected to long-lasting epigenetic regulation, as implied by the previous observation that some $CXCR5^+$ T cells do not completely lose CXCR5 expression a month after being transferred into antigen-free hosts (Lüthje et al., 2012).

While how BCL6 precisely controls STIM1 and PLCγ1 expression and promotes calcium signaling in Tfh cells remains to be further investigated, it is notable that BCL6 expression in T cells partly depends on the nuclear factor of activated T cell (NFAT) transcription factor (Martinez et al., 2016; Vaeth et al., 2016), which is activated by calcium signaling (Gwack et al., 2007). This pathway potentially explains how the amount of BCL6 protein in normal Tfh cells can rapidly increase in response

CellPress
OPEN ACCESS

to acute antigen stimulation *in vivo*. A positive feedback might operate inside Tfh cells between BCL6-promoted calcium signaling downstream of TCR stimulation and NFAT-facilitated BCL6 expression. When BCL6 becomes insufficient, even though the calcium signaling is not totally abolished, a reduced efficiency in T-B cell interactions would compromise help delivery, hamper Tfh cell maintenance, and reduce GC formation. In this scenario, development and maintenance of full Tfh cell features require intrinsic BCL6 expression but invoke both cell-autonomous and non-autonomous modes of action.

The role of BCL6 in regulating calcium signaling and T-B cell interactions is reminiscent of ICOS, which co-stimulates TCR-mediated calcium signaling and thereby promotes T-B cell interactions (Liu et al., 2015). It is also reminiscent of SAP, which protects proximal TCR signaling (Chu et al., 2014; Kageyama et al., 2012; Qi, 2012) and thereby promotes T-B cell interactions (Qi et al., 2008). It is probably not a coincidence that these three T cell-expressed factors essential for GC formation all impinge on antigen-triggered signaling, all regulate physical T-B cell interactions, and none demonstrably instruct a unique signaling pathway dedicated for Tfh cell development or maintenance, with the potential exception for ICOS-TBK1-associated signaling (Pedros et al., 2016), perhaps because sufficient CD40L delivery through physical T-B cell contacts is likely the only help factor that is absolutely essential and irreplaceable for GC formation in a T-dependent B cell response (Wan et al., 2019).

Limitations of study

Our multiple attempts to rescue the helper activity of BCL6-insufficient T cells with STIM1 overexpression did not yield consistent results, potentially because of strict requirement for appropriate stoichiometry of STIM and ORAI proteins, which is difficult to achieve using retroviral overexpression. The current study has not established a causal link between reduced STIM1 and/or PLC-γ1 expression and defective T-B cell interactions when T cells are BCL6 insufficient.

STAR★METHODS

Detailed methods are provided in the online version of this paper and include the following:

- KEY RESOURCES TABLE
- RESOURCE AVAILABILITY
  - Lead contact
  - Materials availability
  - Data and code availability
- EXPERIMENTAL MODEL AND SUBJECT DETAILS
  - Animal experimental models
- METHOD DETAILS
  - Cell **isolation, culture and retroviral** transduction
  - Adoptive transfer, immunization, and perturbation of T-B cell interactions
  - Flow cytometry
  - Immunohistochemistry
  - CD40L mobilization of T cells *ex vivo*
  - Intravital imaging
  - RNA-seq and Gene Set Enrichment Analysis (GSEA)
  - Statistical analysis

SUPPLEMENTAL INFORMATION

Supplemental information can be found online at https://doi.org/10.1016/j.immuni.2021.08.003.

ACKNOWLEDGMENTS

The authors thank Drs. L. Staudt and P. Schwartzberg for providing the germline *Bcl6*$^{-/-}$ and *Sap*$^{-/-}$ mice, respectively; Dr. T. Takemori for providing *Bcl6*$^{fl/fl}$ mice; and Dr. M. Nussenzweig for providing the αDEC-OVA reagent. This work was supported in part by the National Key R&D Program of China (Ministry of Science and Technology, 2018YFE0200300), the Natural Science Foundation of China (8162002 and 81761128019), and the Beijing Municipal Science and Technology Commission. H.Q. is an HHMI International Research Scholar, and this work was also funded in part by the Bill & Melinda Gates Foundation and the Howard Hughes Medical Institute. The findings and conclusions within are those of the authors and do not necessarily reflect positions or policies of the Bill & Melinda Gates Foundation or the Howard Hughes Medical Institute.

AUTHOR CONTRIBUTIONS

H.Q. conceptualized and supervised the study. D.L., J.Y., and J.S. conducted a majority of experiments. B.L. and X.S. conducted imaging and animal experiments, Y.L. and J.S. conducted RNA-seq analysis. H.Q. wrote the paper together with D.L. and B.L. All authors contributed to data interpretation.

DECLARATION OF INTERESTS

The authors declare no competing interests.

Received: April 12, 2020
Revised: June 15, 2021
Accepted: August 4, 2021
Published: August 30, 2021

REFERENCES

Arnold, C.N., Campbell, D.J., Lipp, M., and Butcher, E.C. (2007). The germinal center response is impaired in the absence of T cell-expressed CXCR5. Eur. J. Immunol. *37*, 100–109.

Banchereau, J., Bazan, F., Blanchard, D., Brière, F., Galizzi, J.P., van Kooten, C., Liu, Y.J., Rousset, F., and Saeland, S. (1994). The CD40 antigen and its ligand. Annu. Rev. Immunol. *12*, 881–922.

Basso, K., Klein, U., Niu, H., Stolovitzky, G.A., Tu, Y., Califano, A., Cattoretti, G., and Dalla-Favera, R. (2004). Tracking CD40 signaling during germinal center development. Blood *104*, 4088–4096.

Baumjohann, D., Preite, S., Reboldi, A., Ronchi, F., Ansel, K.M., Lanzavecchia, A., and Sallusto, F. (2013). Persistent antigen and germinal center B cells sustain T follicular helper cell responses and phenotype. Immunity *38*, 596–605.

Berek, C., Berger, A., and Apel, M. (1991). Maturation of the immune response in germinal centers. Cell *67*, 1121–1129.

Campbell, D.J., Kim, C.H., and Butcher, E.C. (2001). Separable effector T cell populations specialized for B cell help or tissue inflammation. Nat. Immunol. *2*, 876–881.

Cannons, J.L., Qi, H., Lu, K.T., Dutta, M., Gomez-Rodriguez, J., Cheng, J., Wakeland, E.K., Germain, R.N., and Schwartzberg, P.L. (2010). Optimal germinal center responses require a multistage T cell:B cell adhesion process involving integrins, SLAM-associated protein, and CD84. Immunity *32*, 253–265.

Cheli, V.T., Santiago González, D.A., Smith, J., Spreuer, V., Murphy, G.G., and Paez, P.M. (2016). L-type voltage-operated calcium channels contribute to astrocyte activation In vitro. Glia *64*, 1396–1415.

Chen, X., Ma, W., Zhang, T., Wu, L., and Qi, H. (2015). Phenotypic Tfh development promoted by CXCR5-controlled re-localization and IL-6 from radiation-resistant cells. Protein Cell *6*, 825–832.

Chu, C., Wang, Y., Zhang, X., Ni, X., Cao, J., Xu, W., Dong, Z., Yuan, P., Wei, W., Ma, Y., et al. (2014). SAP-regulated T Cell-APC adhesion and ligation-dependent and -independent Ly108-CD3ζ interactions. J. Immunol. *193*, 3860–3871.

Coico, R.F., Bhogal, B.S., and Thorbecke, G.J. (1983). Relationship of germinal centers in lymphoid tissue to immunologic memory. VI. Transfer of B cell memory with lymph node cells fractionated according to their receptors for peanut agglutinin. J. Immunol. *131*, 2254–2257.

Crotty, S. (2011). Follicular helper CD4 T cells (TFH). Annu. Rev. Immunol. *29*, 621–663.

Crotty, S. (2014). T follicular helper cell differentiation, function, and roles in disease. Immunity *41*, 529–542.

Cyster, J.G. (2010). B cell follicles and antigen encounters of the third kind. Nat. Immunol. *11*, 989–996.

Czar, M.J., Kersh, E.N., Mijares, L.A., Lanier, G., Lewis, J., Yap, G., Chen, A., Sher, A., Duckett, C.S., Ahmed, R., and Schwartzberg, P.L. (2001). Altered lymphocyte responses and cytokine production in mice deficient in the X-linked lymphoproliferative disease gene SH2D1A/DSHP/SAP. Proc. Natl. Acad. Sci. USA *98*, 7449–7454.

Deenick, E.K., Chan, A., Ma, C.S., Gatto, D., Schwartzberg, P.L., Brink, R., and Tangye, S.G. (2010). Follicular helper T cell differentiation requires continuous antigen presentation that is independent of unique B cell signaling. Immunity *33*, 241–253.

Dent, A.L., Shaffer, A.L., Yu, X., Allman, D., and Staudt, L.M. (1997). Control of inflammation, cytokine expression, and germinal center formation by BCL-6. Science *276*, 589–592.

Förster, R., Mattis, A.E., Kremmer, E., Wolf, E., Brem, G., and Lipp, M. (1996). A putative chemokine receptor, BLR1, directs B cell migration to defined lymphoid organs and specific anatomic compartments of the spleen. Cell *87*, 1037–1047.

Garside, P., Ingulli, E., Merica, R.R., Johnson, J.G., Noelle, R.J., and Jenkins, M.K. (1998). Visualization of specific B and T lymphocyte interactions in the lymph node. Science *281*, 96–99.

Gunn, M.D., Ngo, V.N., Ansel, K.M., Ekland, E.H., Cyster, J.G., and Williams, L.T. (1998). A B-cell-homing chemokine made in lymphoid follicles activates Burkitt's lymphoma receptor-1. Nature *391*, 799–803.

Gwack, Y., Feske, S., Srikanth, S., Hogan, P.G., and Rao, A. (2007). Signalling to transcription: store-operated Ca2+ entry and NFAT activation in lymphocytes. Cell Calcium *42*, 145–156.

Hatzi, K., Nance, J.P., Kroenke, M.A., Bothwell, M., Haddad, E.K., Melnick, A., and Crotty, S. (2015). BCL6 orchestrates Tfh cell differentiation via multiple distinct mechanisms. J. Exp. Med. *212*, 539–553.

Haynes, N.M., Allen, C.D., Lesley, R., Ansel, K.M., Killeen, N., and Cyster, J.G. (2007). Role of CXCR5 and CCR7 in follicular Th cell positioning and appearance of a programmed cell death gene-1^{high} germinal center-associated subpopulation. J. Immunol. *179*, 5099–5108.

Horikawa, K., Yamada, Y., Matsuda, T., Kobayashi, K., Hashimoto, M., Matsuura, T., Miyawaki, A., Michikawa, T., Mikoshiba, K., and Nagai, T. (2010). Spontaneous network activity visualized by ultrasensitive Ca$^{(2+)}$ indicators, yellow Cameleon-Nano. Nat. Methods *7*, 729–732.

Jacob, J., Kelsoe, G., Rajewsky, K., and Weiss, U. (1991). Intraclonal generation of antibody mutants in germinal centres. Nature *354*, 389–392.

Johnston, R.J., Poholek, A.C., DiToro, D., Yusuf, I., Eto, D., Barnett, B., Dent, A.L., Craft, J., and Crotty, S. (2009). Bcl6 and Blimp-1 are reciprocal and antagonistic regulators of T follicular helper cell differentiation. Science *325*, 1006–1010.

Kageyama, R., Cannons, J.L., Zhao, F., Yusuf, I., Lao, C., Locci, M., Schwartzberg, P.L., and Crotty, S. (2012). The receptor Ly108 functions as a SAP adaptor-dependent on-off switch for T cell help to B cells and NKT cell development. Immunity *36*, 986–1002.

Kaji, T., Ishige, A., Hikida, M., Taka, J., Hijikata, A., Kubo, M., Nagashima, T., Takahashi, Y., Kurosaki, T., Okada, M., et al. (2012). Distinct cellular pathways select germline-encoded and somatically mutated antibodies into immunological memory. J. Exp. Med. *209*, 2079–2097.

Kerfoot, S.M., Yaari, G., Patel, J.R., Johnson, K.L., Gonzalez, D.G., Kleinstein, S.H., and Haberman, A.M. (2011). Germinal center B cell and T follicular helper cell development initiates in the interfollicular zone. Immunity *34*, 947–960.

Koguchi, Y., Thauland, T.J., Slifka, M.K., and Parker, D.C. (2007). Preformed CD40 ligand exists in secretory lysosomes in effector and memory CD4+ T cells and is quickly expressed on the cell surface in an antigen-specific manner. Blood *110*, 2520–2527.

Koguchi, Y., Buenafe, A.C., Thauland, T.J., Gardell, J.L., Bivins-Smith, E.R., Jacoby, D.B., Slifka, M.K., and Parker, D.C. (2012). Preformed CD40L is stored in Th1, Th2, Th17, and T follicular helper cells as well as CD4+ 8- thymocytes and invariant NKT cells but not in Treg cells. PLoS ONE *7*, e31296.

Kroenke, M.A., Eto, D., Locci, M., Cho, M., Davidson, T., Haddad, E.K., and Crotty, S. (2012). Bcl6 and Maf cooperate to instruct human follicular helper CD4 T cell differentiation. J. Immunol. *188*, 3734–3744.

Li, F., Zeng, Z., Xing, S., Gullicksrud, J.A., Shan, Q., Choi, J., Badovinac, V.P., Crotty, S., Peng, W., and Xue, H.H. (2018). Ezh2 programs T_{FH} differentiation by integrating phosphorylation-dependent activation of Bcl6 and polycomb-dependent repression of p19Arf. Nat. Commun. *9*, 5452.

Liu, X., Yan, X., Zhong, B., Nurieva, R.I., Wang, A., Wang, X., Martin-Orozco, N., Wang, Y., Chang, S.H., Esplugues, E., et al. (2012). Bcl6 expression specifies the T follicular helper cell program in vivo. J Exp Med *209*, 1841–1852, S1841-1824.

Liu, X., Chen, X., Zhong, B., Wang, A., Wang, X., Chu, F., Nurieva, R.I., Yan, X., Chen, P., van der Flier, L.G., et al. (2014). Transcription factor achaete-scute homologue 2 initiates follicular T-helper-cell development. Nature *507*, 513–518.

Liu, D., Xu, H., Shih, C., Wan, Z., Ma, X., Ma, W., Luo, D., and Qi, H. (2015). T-B-cell entanglement and ICOSL-driven feed-forward regulation of germinal centre reaction. Nature *517*, 214–218.

Liu, X., Lu, H., Chen, T., Nallaparaju, K.C., Yan, X., Tanaka, S., Ichiyama, K., Zhang, X., Zhang, L., Wen, X., et al. (2016a). Genome-wide Analysis Identifies Bcl6-Controlled Regulatory Networks during T Follicular Helper Cell Differentiation. Cell Rep. *14*, 1735–1747.

Liu, X., Lu, H., Chen, T., Nallaparaju, K.C., Yan, X., Tanaka, S., Ichiyama, K., Zhang, X., Zhang, L., Wen, X., et al. (2016b). Genome-wide Analysis Identifies Bcl6-Controlled Regulatory Networks during T Follicular Helper Cell Differentiation. Cell Rep. *14*, 1735–1747.

Lu, K.T., Kanno, Y., Cannons, J.L., Handon, R., Bible, P., Elkahloun, A.G., Anderson, S.M., Wei, L., Sun, H., O'Shea, J.J., and Schwartzberg, P.L. (2011). Functional and epigenetic studies reveal multistep differentiation and plasticity of in vitro-generated and in vivo-derived follicular T helper cells. Immunity *35*, 622–632.

Lüthje, K., Kallies, A., Shimohakamada, Y., Belz, G.T., Light, A., Tarlinton, D.M., and Nutt, S.L. (2012). The development and fate of follicular helper T cells defined by an IL-21 reporter mouse. Nat. Immunol. *13*, 491–498.

MacLennan, I.C. (1994). Germinal centers. Annu. Rev. Immunol. *12*, 117–139.

Martinez, G.J., Hu, J.K., Pereira, R.M., Crampton, J.S., Togher, S., Bild, N., Crotty, S., and Rao, A. (2016). Cutting Edge: NFAT Transcription Factors Promote the Generation of Follicular Helper T Cells in Response to Acute Viral Infection. J. Immunol. *196*, 2015–2019.

Nurieva, R.I., Chung, Y., Martinez, G.J., Yang, X.O., Tanaka, S., Matskevitch, T.D., Wang, Y.H., and Dong, C. (2009). Bcl6 mediates the development of T follicular helper cells. Science *325*, 1001–1005.

Okada, T., Miller, M.J., Parker, I., Krummel, M.F., Neighbors, M., Hartley, S.B., O'Garra, A., Cahalan, M.D., and Cyster, J.G. (2005). Antigen-engaged B cells undergo chemotaxis toward the T zone and form motile conjugates with helper T cells. PLoS Biol. *3*, e150.

Pedros, C., Zhang, Y., Hu, J.K., Choi, Y.S., Canonigo-Balancio, A.J., Yates, J.R., 3rd, Altman, A., Crotty, S., and Kong, K.F. (2016). A TRAF-like motif of the inducible costimulator ICOS controls development of germinal center TFH cells via the kinase TBK1. Nat. Immunol. *17*, 825–833.

Qi, H. (2012). From SAP-less T cells to helpless B cells and back: dynamic T-B cell interactions underlie germinal center development and function. Immunol. Rev. *247*, 24–35.

CellPress
OPEN ACCESS

Qi, H. (2016). T follicular helper cells in space-time. Nat. Rev. Immunol. *16*, 612–625.

Qi, H., Cannons, J.L., Klauschen, F., Schwartzberg, P.L., and Germain, R.N. (2008). SAP-controlled T-B cell interactions underlie germinal centre formation. Nature *455*, 764–769.

Qi, H., Liu, D., Ma, W., Wang, Y., and Yan, H. (2014). Bcl-6 controlled TFH polarization and memory: the known unknowns. Curr. Opin. Immunol. *28*, 34–41.

Schaerli, P., Willimann, K., Lang, A.B., Lipp, M., Loetscher, P., and Moser, B. (2000). CXC chemokine receptor 5 expression defines follicular homing T cells with B cell helper function. J. Exp. Med. *192*, 1553–1562.

Shaw, L.A., Bélanger, S., Omilusik, K.D., Cho, S., Scott-Browne, J.P., Nance, J.P., Goulding, J., Lasorella, A., Lu, L.F., Crotty, S., and Goldrath, A.W. (2016). Id2 reinforces TH1 differentiation and inhibits E2A to repress TFH differentiation. Nat. Immunol. *17*, 834–843.

Shulman, Z., Gitlin, A.D., Weinstein, J.S., Lainez, B., Esplugues, E., Flavell, R.A., Craft, J.E., and Nussenzweig, M.C. (2014). Dynamic signaling by T follicular helper cells during germinal center B cell selection. Science *345*, 1058–1062.

Vaeth, M., Eckstein, M., Shaw, P.J., Kozhaya, L., Yang, J., Berberich-Siebelt, F., Clancy, R., Unutmaz, D., and Feske, S. (2016). Store-Operated Ca(2+) Entry in Follicular T Cells Controls Humoral Immune Responses and Autoimmunity. Immunity *44*, 1350–1364.

Victora, G.D., and Nussenzweig, M.C. (2012). Germinal centers. Annu. Rev. Immunol. *30*, 429–457.

Victora, G.D., Schwickert, T.A., Fooksman, D.R., Kamphorst, A.O., Meyer-Hermann, M., Dustin, M.L., and Nussenzweig, M.C. (2010). Germinal center dynamics revealed by multiphoton microscopy with a photoactivatable fluorescent reporter. Cell *143*, 592–605.

Vinuesa, C.G., and Cyster, J.G. (2011). How T cells earn the follicular rite of passage. Immunity *35*, 671–680.

Vinuesa, C.G., Linterman, M.A., Yu, D., and MacLennan, I.C. (2016a). Follicular Helper T Cells. Annu. Rev. Immunol. *34*, 335–368.

Vinuesa, C.G., Linterman, M.A., Yu, D., and MacLennan, I.C. (2016b). Follicular Helper T Cells. Annu. Rev. Immunol. *34*, 335–368.

Wan, Z., Lin, Y., Zhao, Y., and Qi, H. (2019). T_{FH} cells in bystander and cognate interactions with B cells. Immunol. Rev. *288*, 28–36.

Xu, H., Li, X., Liu, D., Li, J., Zhang, X., Chen, X., Hou, S., Peng, L., Xu, C., Liu, W., et al. (2013). Follicular T-helper cell recruitment governed by bystander B cells and ICOS-driven motility. Nature *496*, 523–527.

Yu, D., Rao, S., Tsai, L.M., Lee, S.K., He, Y., Sutcliffe, E.L., Srivastava, M., Linterman, M., Zheng, L., Simpson, N., et al. (2009). The transcriptional repressor Bcl-6 directs T follicular helper cell lineage commitment. Immunity *31*, 457–468.

STAR★METHODS

KEY RESOURCES TABLE

REAGENT or RESOURCE	SOURCE	IDENTIFIER
Antibodies		
anti-CD19 APC-Cy7 (clone 1D3)	eBioscience	CAT# 25-0193-82
anti-B220 APC-Cy7 (clone RA3-6B2)	eBioscience	CAT# 47-0452-82
anti-CD4 AF700 (clone RM 4-5)	eBioscience	CAT# 56-0042-82
anti-CD4 APC (clone GK1.5)	eBioscience	CAT# 17-0041-82
anti-CD4 FITC (clone GK1.5)	eBioscience	CAT# 11-0041-82
anti-CD44 AF700 (clone 1M7)	eBioscience	CAT# 56-0441-82
anti-CD45.1 APC (clone A20)	eBioscience	CAT# 17-0453-82
anti-CD45.2 eFlour450 (clone 104)	eBioscience	CAT# 48-0454-82
anti-CD95 PE-Cy7 (clone Jo2)	BD Biosciences	CAT# 557653
anti-GL7 eFlour450 (clone GL7)	eBioscience	CAT# 48-5902-82
anti-PD-1 PE-Cy7 (clone RMP1-30)	Biolegend	CAT# 109110
anti-IgM^{a} PE (clone DS-1)	BD Biosciences	CAT# 553517
anti-IgD FITC (clone 11-26c)	eBioscience	CAT# 11-5993-82
anti-CXCR5 biotinylated (clone 2G8)	BD Biosciences	CAT# 551960
anti-CD40L PE (clone MR1)	BD Biosciences	CAT# 553658
Streptavidin PE	BD Biosciences	CAT# 554061
Streptavidin APC	BioLegend	CAT# 554067
NP-PE	Biosearch Technologies	CAT# N-5070-1
7-AAD	Biotium	CAT# 40084
Anti-CD40L (clone MR1)	Bio X Cell	CAT# BP0017-1
Anti-CD3ε (clone 145-2C11)	Bio X Cell	CAT# BP0001-1
Anti-CD28 (clone 37.51)	Bio X Cell	CAT# BE0015-1
Commercial reagents		
Lipopolysaccharides (*Escherichia coli*, serotype O111:B4)	Sigma-Aldrich	CAT# L2630-25MG
NP-OVA	Biosearch Technologies	CAT# N-5051-10
NP-KLH	Biosearch Technologies	CAT# N-5060
Imject Alum Adjuvant	Thermo Fisher	CAT# 77161
Lysozyme from chicken egg white	Sigma-Aldrich	CAT# L4919
Albumin from chicken egg white	Sigma-Aldrich	CAT# A2512
RNase Inhibitor	Takara	CAT# 2313B
TE buffer	Invitrogen	CAT# 12090015
Triton X-100	Invitrogen	CAT# 15596018
Oligo-dT	Takara	CAT# 3806
dNTP	Invitrogen	CAT# 18427013
SuperScript II reverse transcriptase	Invitrogen	CAT# 18064071
Superscript II first-strand buffer	Invitrogen	CAT# 18064014
DTT	Solarbio	CAT# D1070-5
Betaine	Sigma	CAT# 107-43-7
MgCl2	Invitrogen	CAT# AM9530G
KAPA HiFi HotStart ReadyMix	KAPA	CAT# Kk2601
DNA clean beads	Vazyme	CAT# N411-02
Nuclease-free water	Invitrogen	CAT# 10977015
TruePrep DNA Library Prep Kit V2 for Illumina	Vazyme	CAT# TD501-02
SDS	Takara	CAT# 3806

(*Continued on next page*)

CellPress
OPEN ACCESS

Continued

REAGENT or RESOURCE	SOURCE	IDENTIFIER
Q5 HF master mix	NEB	CAT# M0492L
Tris	Amresco	N/A
Critical commercial assays		
Cytoperm/Cytofix kit	BD Biosciences	CAT# 554714
Foxp3/Transcription Factor Staining Buffer Set	eBioscience	CAT# 00-5523-00
Deposited data		
mRNA sequencing data of pre-GC B cells helped by *Cd4-cre* or *Cd4-cre* × *Bcl6*$^{fl/+}$ T cells	This paper	GSE181081
mRNA sequencing data of activated *Cd4-cre* or *Cd4-cre* × *Bcl6*$^{fl/+}$ T cells	This paper	GSE181081
Recombinant DNA		
Plasmid pMSCV		N/A
Plasmid pIE		N/A
Plasmid for DEC205-OVA	Gift from Dr. M. Nussenzweig	N/A
Plasmid for YC-nano50^{CD} reporter		N/A
Experimental models: Organisms/strains		
Mouse: C57BL/6J		Jax 664
Mouse: *Sap*$^{-/-}$	Gift from Dr. P. Schwartzberg (Czar et al., 2001)	N/A
Mouse: *Bcl6*$^{-/-}$	Gift from Dr. L. Staudt (Dent et al., 1997)	N/A
Mouse: *Bcl6*$^{fl/fl}$	Gift from Dr. T. Takemori (Kaji et al., 2012)	N/A
Mouse: CD45.1		Jax 002014
Mouse: CD4-Cre		Jax 022071
Mouse: GFP-expressing		Jax 4353
Mouse: CFP-expressing		Jax 4218
Mouse: dsRed-expressing		Jax 6051
Mouse: OT-II		Jax 4194
Mouse: MD4		Jax 2595
Software and algorithms		
FlowJo v10	Tree Star	N/A
ImageJ	Wayne Rasband	N/A
Imaris v9	Bitplane	N/A
Prism v6	Graphpad	N/A
GSEA	Broad Institute	N/A
Adobe AfterEffect	Adobe	N/A
Adobe PhotoShop	Adobe	N/A
FastQC		v0.11.9
Hisat2		version 2.2.1
Samtools		version 1.10
HTSeq-count		version 0.12.4
DESeq2 software	R	N/A
Other		
HiSeq × Ten sequencer	Illumina	N/A

RESOURCE AVAILABILITY

Lead contact

Further information and requests for resources and reagents should be directed and will be fulfilled by the lead contact Hai Qi (qihai@tsinghua.edu.cn).

Materials availability

Supply of the following reagents and mice are subject to MTA agreements: DEC205-OVA (Dr. M. Nussenzweig); *Bcl6*$^{-/-}$ mice (Dr. L. Staudt); *Bcl6*$^{fl/fl}$ mice (Dr. T. Takemori).

Data and code availability

mRNA sequencing data have been deposited at GEO and are publicly available as of the date of publication. Accession numbers are listed in the key resources table. Microscopy data reported in this paper will be shared by the lead contact upon request. Any additional information required to reanalyze the data reported in this paper is available from the lead contact upon request.

EXPERIMENTAL MODEL AND SUBJECT DETAILS

Animal experimental models

C57BL/6 (Jax 664), CD45.1 (Jax 002014), CD4-Cre (Jax 022071), GFP-expressing (Jax 4353), CFP-expressing (Jax 4218), dsRed-expressing (Jax 6051), $OVA_{323\text{-}339}$-specific TCR transgenic OT-II (Jax 4194) and HEL-specific Ig-transgenic MD4 (Jax 2595) mice were originally purchased from the Jackson Laboratory. *Bcl6*$^{-/-}$ mice (Dent et al., 1997), *Bcl6*$^{fl/fl}$ mice (Kaji et al., 2012) and *Sap*$^{-/-}$ mice (Czar et al., 2001) were gifts from Drs. L. Staudt, T. Takemori and P. Schwartzberg, respectively. Relevant alleles were interbred to obtain desired genotypes. All mice were maintained under specific-pathogen free conditions, and used according to institutional and governmental guidelines for animal welfare.

METHOD DETAILS

Cell isolation, culture and retroviral transduction

Naive OT-II T cells, polyclonal T cells, MD4 B cells or polyclonal B cells were isolated using the negative CD4 T cell or B cell isolation kit (Miltenyi Biotec). To obtain activated T cells *in vitro*, naive OT-II or polyclonal B6 CD4 T cells were cultivated on plates coated with anti-CD3 (8 μg/mL) and anti-CD28 (8 μg/mL). Retroviral vectors that express relevant target genes were packaged with the Plat-E system, as previously described (Xu et al., 2013). For transduction, activated T cells were spin-infected at 2200 rpm with viral supernatants in the presence of 1 μg/mL polybrene (Sigma) for 2 h at 32°C. Infected T cells were then cultivated in the presence of 10 ng/mL human IL-2 until use for *in vitro* experiments or for adoptive transfer.

Adoptive transfer, immunization, and perturbation of T-B cell interactions

For various *in vivo* experiments, naive or *in vitro* activated and retrovirally transduced OT-II T cells were intravenously injected into B6 or SAP-deficient mice, alone or in combination with MD4 B cells. For certain experiments, total splenocytes were intravenously injected into sub-lethally irradiated CD45.1 SAP-deficient mice. In such experiments, the number of CD4 T cells in the splenocyte inoculum was kept constant among different groups. Typically, splenocytes from one donor mouse were split and transferred into three recipients. For subcutaneous immunization, each recipient mouse was given either 30 μg NP-OVA (Biosearch Technology) or 30 μg HEL-OVA antigen plus 0.5 μg LPS in alum (Thermo Scientific). For intraperitoneal immunization, each recipient mouse was given 100 μg NP-OVA (Biosearch Technology) or 100 μg NP-KLH (Biosearch Technologies) or 50 μg HEL-OVA antigen plus 1 μg LPS in alum (Thermo Scientific). For CD40L blockade in the early follicular phase, HEL-OVA-immunized B6 recipients of OT-II T cells and MD4 B cells were intravenously given 200 μg αCD40L antibody or PBS 50-60 h post immunization. For αDEC-OVA and CD40L blockade experiments, HEL-OVA-immunized B6 recipients of OT-II T cells and MD4 B cells were intravenously given 50 μg αDEC-OVA or 100 μg αCD40L antibody or PBS 100 h post immunization, a time point well into the follicular phase of the response.

Flow cytometry

Single-cell suspension of splenic or lymph node cells were incubated with 10% 2.4G2 culture supernatants to block Fc receptors and then stained with indicated antibodies in MACS buffer (PBS supplemented with 1% FBS and 5mM EDTA). Staining reagents include: APC-Cy7 anti-CD19 (1D3), APC-Cy7 anti-B220 (RA3-6B2), AF700 anti-CD4 (RM 4-5), APC anti-CD4 (GK1.5), FITC anti-CD4 (gk1.5), AF700 anti-CD44 (IM7), APC anti-CD45.1 (A20), eF450 anti-CD45.2 (104), PE-Cy7 anti-CD95 (Jo2), eFlour450 anti-GL7 (GL7), PE-Cy7 anti-PD-1 (RMP1-30), PE anti-IgM^{a} (DS-1), FITC anti-IgD (11-26c) from eBioscience; biotinylated anti-CXCR5 (2G8), PE anti-CD40L (MR1), streptavidin PE (Cat 554061) and streptavidin APC (Cat 554067) from BD Biosciences. Dead-cell exclusion was based on 7-AAD staining (Biotium), and non-singlet events were excluded with FSC-H/FSC-W and SSC-H/SSC-W. Isotype-matched non-specific antibodies were purchased from the corresponding companies. Cells were stained with primary antibodies for 60-90 min, washed, and then with secondary reagents for 30 min on ice. For intracellular staining, cells were stained using Cytoperm/Cytofix kit (BD Biosciences) according to the manufacturer's protocol. All flow-cytometry data were collected on an LSR-II or Aria III (BD).

Immunohistochemistry

To examine T cell distribution in the draining lymph node, tissues were fixed with paraformaldehyde, and sections were stained with EF450-anti-IgD and APC-anti-CD3, mounted with the ProlongGold Antifade reagent (Invitrogen), and imaged with an Olympus FV1000 upright microscopy using 20 × lens.

CD40L mobilization of T cells *ex vivo*

As described previously (Liu et al., 2015), OT-II T cells of indicated genotypes were transferred into B6 mice, which were subsequently immunized with NP-OVA intraperitoneally. 7 to 8 days later, splenocytes were stimulated by anti-CD3 (2 μg/mL) for 15 min in the presence of an anti-CD40L staining antibody (clone MR1) to capture externalized CD40L on the cell surface. Cells were further stained for additional surface markers before analyzed by flow cytometry.

Intravital imaging

Intravital two-photon imaging of mouse inguinal lymph nodes were done as described previously (Liu et al., 2015), including measurement of T-B cell contact extents by contact duration and SEI. To visualize T cell calcium signaling during T-B cell contacts *in vivo*, YC-nano50^{CD} reporter was transduced into *Bcl6*$^{+/+}$ or *Bcl6*$^{+/-}$ OT-II T cells. Ratiometric images of calcium signals were analyzed with the RatioPlus plugin of ImageJ. Quantitative analyses were conducted by the method established previously (Liu et al., 2015). Adobe Photoshop and AfterEffect were used to annotate and prepare image sequences and to make playback videos.

RNA-seq and Gene Set Enrichment Analysis (GSEA)

CD4-Cre × *Bcl6*$^{fl/+}$ or control CD4-Cre OT-II T cells were transferred into CD45.1 *Sap*$^{-/-}$ mice (5 × 10^5 per recipient), which were subcutaneously immunized with 40 μg NP-OVA plus 0.4 μg LPS in alum one day after the cell transfer. At day 3 post immunization, CXCR5^{hi}PD-1^{hi} Tfh cells were sort-purified from the draining lymph node and prepared for RNA-seq analysis. Two technical repeats of ~200 cells per sample from each of 3 mice were included. To analyze NP^{+}IgD^{low}B220^{+} pre-GC B cells, cells from recipients of the same group were pooled to sort-purify 4 200-cell repeats. The SMART-Seq2 protocol for single-cell analysis was adapted and optimized for analyzing ~200 cells. All libraries were sequenced on a HiSeq × Ten sequencer (Illumina). After pre-processed using FastQC (v0.11.9), raw sequences were aligned to the *Mus musculus* reference genome (GRCm38) using Hisat2 (version 2.2.1) and then sorted with Samtools (version 1.10). Reads were counted in genes with the utilization of HTSeq -count (version 0.12.4). Gene expression was calculated with the DESeq2 software in R. Heatmaps and volcano plots were generated with pheatmap and ggplot2 R functions, respectively. GSEA analysis was carried out using the GSEA software from the Broad Institute.

Statistical analysis

Statistics and graphing were done with Prism (Graphpad). Unless indicated otherwise, two-tailed Student's t test was used to compare end-point means of different groups.

Immunity, Volume *54*

Supplemental information

BCL6 controls contact-dependent help delivery during follicular T-B cell interactions

Dan Liu, Jiacong Yan, Jiahui Sun, Bo Liu, Weiwei Ma, Ye Li, Xingxing Shao, and Hai Qi

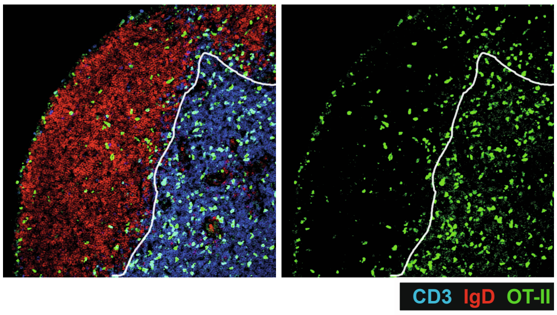

$$\text{Follicle Recruitment Index (FRI)} = \frac{\text{T-cell density in the follicle}}{\text{T-cell density in the T-zone}}$$

Figure S1. Definition of follicle recruitment index (FRI), related to Figure 1.

The follicle recruitment index (FRI) to quantify the efficiency of OT-II follicular localization. The experimental system and color code as in Figure 1B.

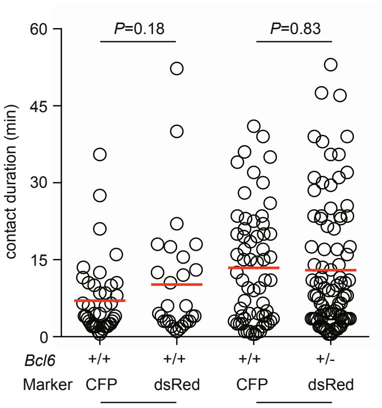

Figure S2. Comparable duration of contacts between MD4 B cells and *Bcl6*$^{+/+}$ or *Bcl6*$^{+/-}$ OT-II T cells at the T-B border, related to Figure 2.

B6 mice that received transfer of GFP-expressing MD4 B cells, control CFP-expressing *Bcl6*$^{+/+}$ and dsRed-expressing test *Bcl6*$^{+/-}$ or test *Bcl6*$^{+/+}$ OT-II T cells were immunized with HEL-OVA. Draining lymph nodes were intravitally imaged 36-48 hours later, a time point at which T-B contacts were mainly seen at the T-B border. Contact durations were quantitated (see corresponding Movie S1 and S2). Each dot represents one contact. Data pooled from two independent imaging experiments, each of which involved at least 2 recipient mice.

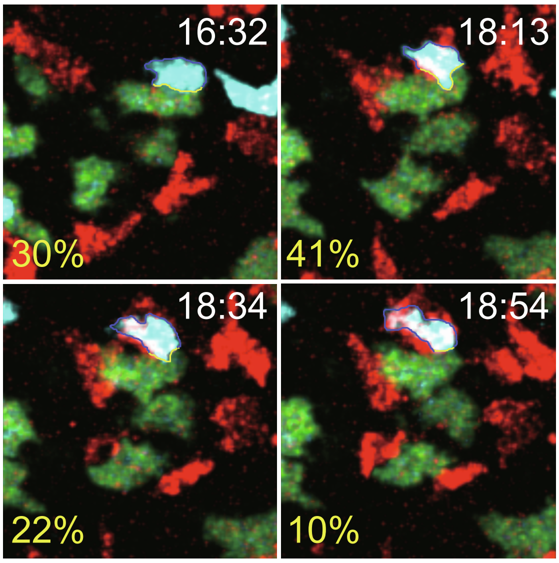

$$\text{SEI} = \max\left(\frac{\text{Length of contact}}{\text{T-cell perimeter}}\right)$$

Figure S3. Definition of surface engagement index (SEI), related to Figure 2.

In maximum-intensity projection, the proportion of T-cell perimeter (blue) that is engaged by an interacting B cell (line fragment in yellow) is traced overtime and the maximum proportion reached during a continuous contact incidence is defined as the surface engagement index.

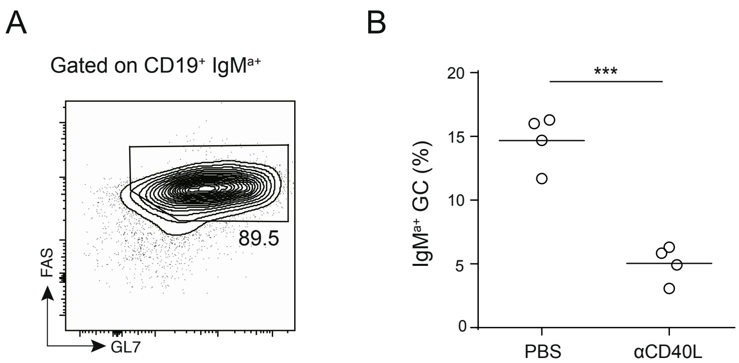

Figure S4. Follicular phase of CD40L delivery is required for GC formation, related to Figure 4.

B6 mice that received transfer of MD4 B cells and OT-II T cells were immunized with HEL-OVA and treated with αCD40L antibody (200 µg) or PBS 50-60 hours later. **A**, Representative FACS profile and frequency of FAS^{hi} $GL7^{hi}$ GC B cells in IgM^{a+} $CD19^{+}$ B cells at 5 days post immunization. **B**, Frequencies of IgM^{a+} MD4 GC cells in total $CD19^{+}$ B cells. Each symbol represents one mouse, and lines denote means. One of three experiments with similar results is shown. *** $P<0.001$.

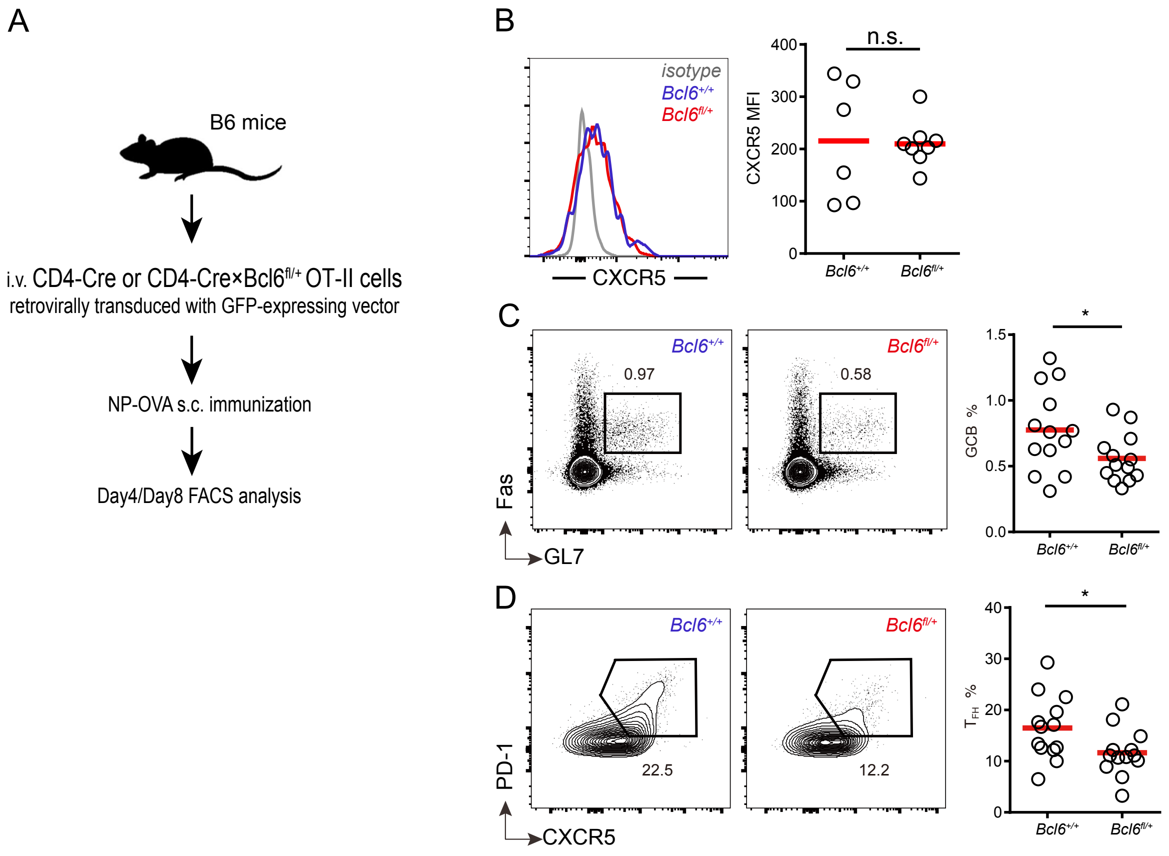

Figure S5. Defective helper functions due to BCL6 haploinsufficiency as demonstrated with T-cell-specific conditional BCL6 deficiency, related to Figure 5.

A, The experimental scheme. **B,** Representative histograms and MFIs of surface CXCR5 expression on OT-II T cells of indicated *Bcl6* genotypes in draining lymph nodes 4 days after NP-OVA immunization. **C,D,** Representative flow-cytometry profiles and frequencies of FAS^{hi}GL7^{hi} GC B cells in total B220^{+} B cells (**C**) or CXCR5^{hi}PD-1^{hi} Tfh cells in OT-II T cells (**D**) 8 days after NP-OVA immunization in CD45.1 *Sap*$^{-/-}$ recipients. Each symbol represents one mouse, and lines denote the means. Data are pooled from 2 independent experiments. n.s., not significant. * P<0.05.

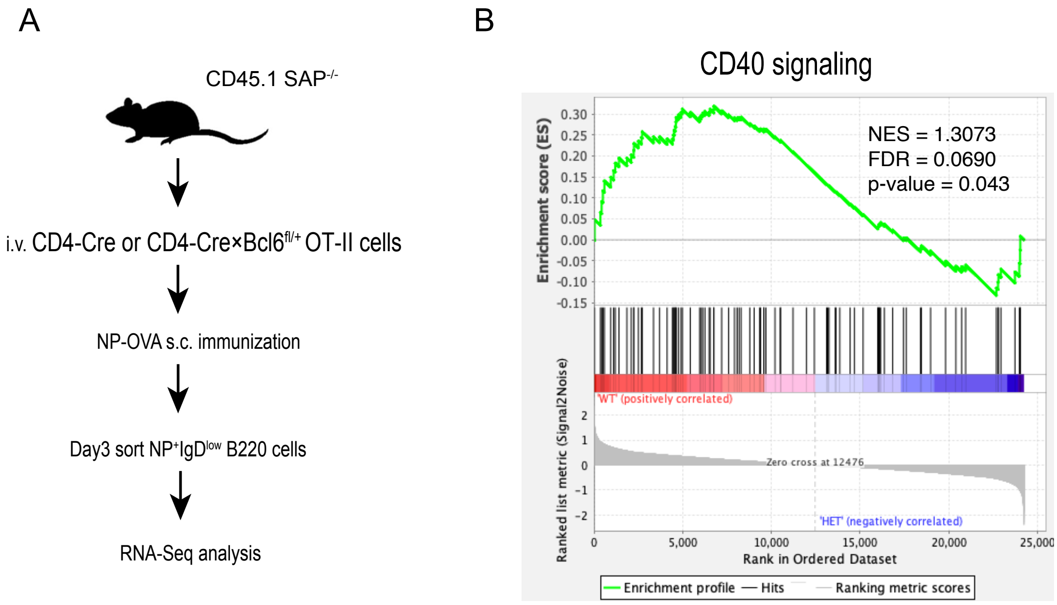

Figure S6. Reduced CD40 signaling signature in antigen-activated B cells helped by BCL6-insufficient T cells, related to Figure 5.

mRNA sequencing of pre-GC B cells helped by CD4-Crex*Bcl6*$^{fl/+}$ or CD4-Cre OT-II T cells. **A,** The experimental scheme. **B,** Enrichment analysis comparing pre-GC antigen-specific B cells helped by CD4-Crex*Bcl6*$^{fl/+}$ ("HET") or by CD4-Cre ("WT") OT-II T cells for a gene set upregulated by CD40L stimulation of Ramos cells previously reported (Basso et al., 2004). NES, normalized enrichment score; FDR, false discovery rate.

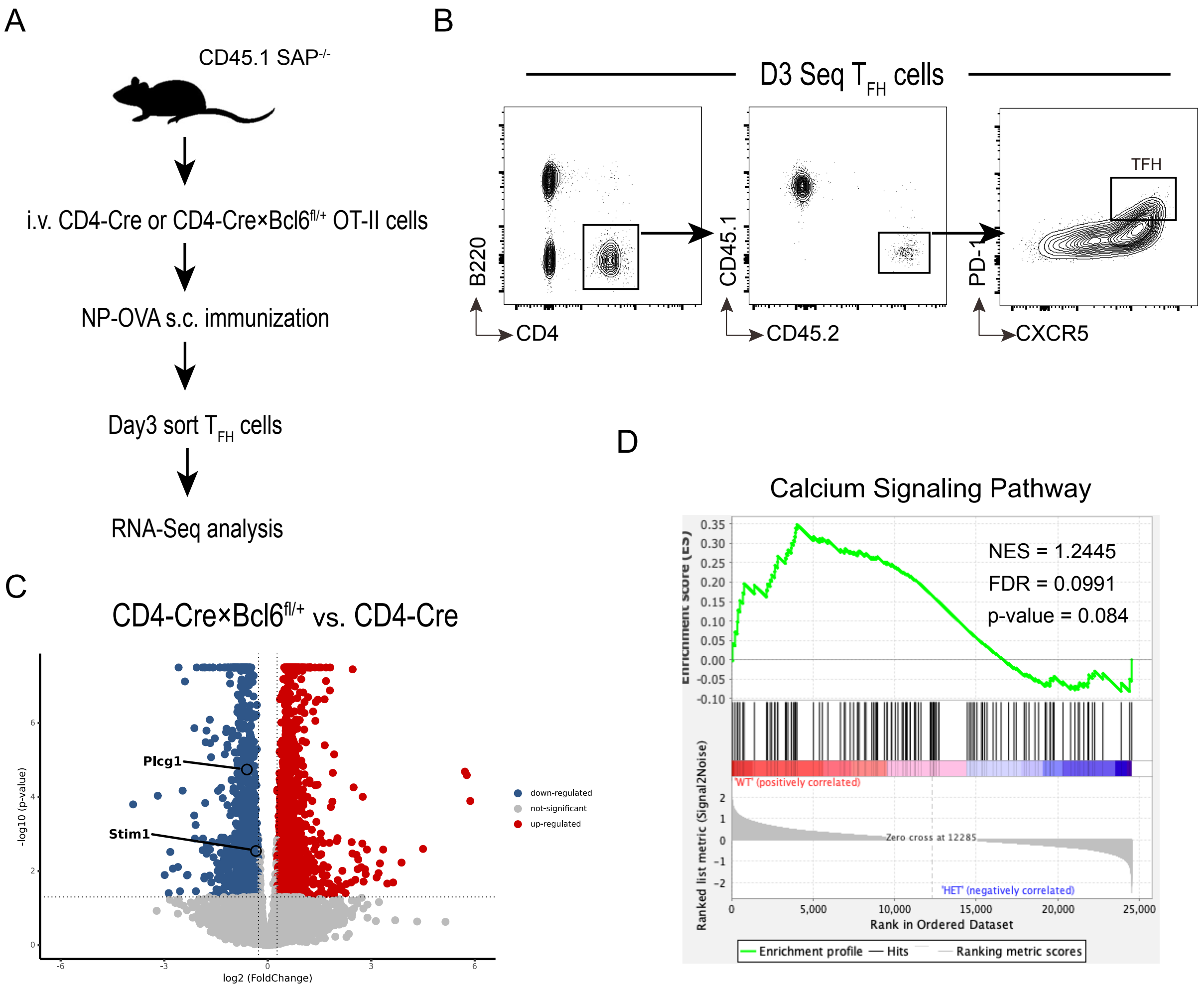

Figure S7. Reduced calcium signaling signature in BCL6-insufficient Tfh cells, related to Figure 3.

mRNA sequencing analysis of CD4-Crex*Bcl6*$^{fl/+}$ or CD4-Cre OT-II Tfh cells 3 days post immunization. **A,** The experimental scheme. **B,** The gating strategy for sorting Tfh cells. **C,** A volcano plot highlighting genes differentially expressed (P<0.05, fold change⩾1.2) between CD4-Crex*Bcl6*$^{fl/+}$ and CD4-Cre Tfh cells. Genes upregulated in CD4-Crex*Bcl6*$^{fl/+}$ Tfh cells are in red, and downregulated are shown in blue. Circles highlight *Stim1* and *Plcg1*. **D,** Enrichment analysis comparing CD4-Crex*Bcl6*$^{fl/+}$ ("HET") or CD4-Cre ("WT") OT-II T cells for genes in the calcium signaling pathway as defined in the KEGG database. NES, normalized enrichment score; FDR, false discovery rate.
